# Dietary Carbohydrate Intake, Carbohydrate Quality, and Healthy Aging in Women

**DOI:** 10.1001/jamanetworkopen.2025.11056

**Published:** 2025-05-16

**Authors:** Andres V. Ardisson Korat, Ecaterina Duscova, M. Kyla Shea, Paul F. Jacques, Paola Sebastiani, Molin Wang, Sara Mahdavi, A. Heather Eliassen, Walter C. Willett, Qi Sun

**Affiliations:** 1 US Department of Agriculture Human Nutrition Research Center on Aging, Tufts University, Boston, Massachusetts; 2 Tufts University School of Medicine, Tufts University, Boston, Massachusetts; 3 Institute for Clinical Research and Health Policy Studies, Tufts Medical Center, Boston, Massachusetts; 4 Department of Epidemiology, Harvard T.H. Chan School of Public Health, Boston, Massachusetts; 5 Channing Division of Network Medicine, Brigham and Women’s Hospital, Boston, Massachusetts; 6 Department of Biostatistics, Harvard T.H. Chan School of Public Health, Boston, Massachusetts; 7 Department of Nutrition, Harvard T.H. Chan School of Public Health, Boston, Massachusetts; 8 Department of Nutritional Sciences, Faculty of Medicine, University of Toronto, Toronto, Ontario, Canada

## Abstract

**Importance:**

Dietary carbohydrate quality is inversely associated with risks of chronic disease and all-cause mortality. However, limited evidence exists regarding the role of carbohydrate quality and dietary carbohydrate types in promoting healthy aging.

**Objective:**

To evaluate the long-term role of dietary carbohydrate intake and carbohydrate quality in healthy aging.

**Design, Setting, and Participants:**

This prospective cohort study used data from the Nurses’ Health Study (NHS) from January 1984 to December 2016 and included participants aged younger than 60 years in 1984. Data were analyzed from January 2023 to February 2025.

**Exposure:**

Intakes of total carbohydrates; refined carbohydrates; high-quality carbohydrates from whole grains, fruits, vegetables, and legumes; dietary fiber; and the dietary glycemic index (GI) and glycemic load (GL).

**Main Outcomes and Measures:**

Nutrient intakes were derived from 1984 and 1986 food frequency questionnaires. The primary outcome was healthy aging, defined as the absence of major chronic diseases, lack of cognitive and physical function impairments, and having good mental health, according to 2014 or 2016 NHS questionnaire data. Multivariate logistic regression was used to calculate associations of each carbohydrate variable with healthy aging.

**Results:**

Among 47 513 participants (mean [SD] baseline age, 48.5 [6.2] years), 3706 (7.8%) met our healthy aging definition. Every 10%-calorie increment in intakes of total carbohydrates (odds ratio [OR], 1.17; 95% CI, 1.10-1.25) and high-quality carbohydrates (OR, 1.31; 95% CI, 1.22-1.41) was positively associated with healthy aging. Refined carbohydrates were associated with lower odds of healthy aging (OR, 0.87; 95% CI, 0.80-0.95). Intakes of carbohydrates from fruits, vegetables, and whole grains were positively associated with odds of healthy aging (ORs ranging from 1.11; 95% CI, 1.07-1.15 to 1.37; 95% CI, 1.20-1.57 per 5% energy increment). Additionally, intakes of total dietary fiber and fiber from fruits, vegetables, and cereals were associated with higher odds of healthy aging (ORs ranging from 1.07; 95% CI, 1.03-1.11 to 1.17; 95% CI, 1.13-1.22 per 1-SD increment). GL was positively associated with healthy aging, which was attenuated by dietary fiber adjustment. A higher GI (OR, 0.76; 95% CI, 0.67-0.87) and carbohydrate-to-fiber ratio (OR, 0.71; 95% CI, 0.62-0.81) were inversely associated with healthy aging when comparing extreme quintiles. There were positive associations for isocaloric replacements of refined carbohydrates, animal protein, total fat, or trans fats with high-quality carbohydrates (ORs ranging from 1.08; 95% CI, 1.01-1.16 to 1.16; 95% CI, 1.11-1.21).

**Conclusions and Relevance:**

In this cohort study of women, intakes of high-quality carbohydrates and dietary fiber were associated with positive health status in older adulthood, suggesting that dietary carbohydrate quality may be an important determinant of healthy aging.

## Key Points

**Question:**

Is dietary carbohydrate intake in midlife prospectively associated with healthy aging considering chronic diseases, physical and cognitive function, and mental health?

**Findings:**

In this cohort study of 47 513 women, intakes of total and high-quality carbohydrates; carbohydrates from whole grains, fruits, vegetables, and legumes; and total dietary fiber in midlife were associated with increased odds of healthy aging and several domains of positive health status in older adulthood. Conversely, refined carbohydrate intake was associated with lower odds of healthy aging.

**Meaning:**

These findings suggest that intakes of dietary fiber, high-quality, and refined carbohydrates may be important determinants of overall health status in older adulthood.

## Teaser

This cohort study assesses associations of dietary carbohydrate intake and carbohydrate quality with healthy aging among participants in the Nurses’ Health Study.

## Introduction

The population of older adults in the US is expected to double in the next 4 decades. Importantly, this group bears a disproportionately large burden of chronic diseases and declining physical and cognitive function.^1,2,3,4^ Diet is an important determinant of chronic diseases,^5,6^ premature death,^7^ physical frailty,^8^ and healthy aging.^9^ Dietary carbohydrate accounts for approximately 50% of energy intake in the US diet.^10^ However, most of it consists of refined carbohydrates (42% of total calories), while only 8% consists of high-quality carbohydrates from whole grains, legumes, fruits, and vegetables.^10^ The association of total carbohydrate intake with all-cause mortality risk follows a U-shaped curve with the lowest risk for 50% to 55% of total calories.^11^ In contrast, intakes of high-quality carbohydrate sources are consistently associated with reduced risk of chronic disease incidence and mortality.^12,13,14^ However, limited evidence exists regarding the role of dietary carbohydrate in the development of healthy aging.^15^

Similarly, the associations of carbohydrate quality indices with healthy aging have not been elucidated. A high carbohydrate-to-fiber ratio has been associated with higher risks of all-cause and cardiovascular disease mortality; however, it has not been evaluated in the context of healthy aging.^16,17,18^ The glycemic index (GI) and glycemic load (GL) reflect the glycemic effects of carbohydrate intake.^19,20^ GI is a relative measure of the incremental blood glucose response per gram of carbohydrate,^21^ and the GL considers the glucose response to both the quantity of carbohydrate intake and its GI.^22^ The association of GI and GL with various health outcomes has been extensively evaluated^20^; however, their role in healthy aging remains inconclusive.^15^ This study aims to comprehensively evaluate the association of dietary carbohydrate quantity and quality assessed in midlife with the likelihood of healthy aging, defined as longevity with no major chronic diseases, good mental health, and no impairments in either cognitive or physical function, in the Nurses’ Health Study (NHS).^23^ We hypothesized that high-quality carbohydrate intake would be positively associated with healthy aging.

## Methods

### Study Population

The protocol for this cohort study was approved by the institutional review boards of Brigham and Women’s Hospital and Tufts University. The reporting follows the Strengthening the Reporting of Observational Studies in Epidemiology (STROBE) reporting guideline. The NHS cohort was established in 1976 with 121 700 female nurses aged 30 to 55 years.^24,25^ Participants provided implied consent by returning completed questionnaires. Participants’ demographic, lifestyle, and health information were collected with biennial questionnaires with more than 90% follow-up. Of 81 702 participants who returned the 1984 questionnaire, we excluded those with a baseline history of any of the 11 chronic diseases included in our healthy aging definition^26,27,28,29,30,31,32^ (Table 1) and participants aged 60 years or older at baseline (eFigure in Supplement 1). We also excluded participants with more than 70 missing items on the baseline food frequency questionnaire (FFQ) and those with implausible energy intake levels (<500 or >3500 kcal/d). Additionally, we excluded participants who did not return the 2016 questionnaire or had missing healthy aging assessments.

**Table 1.** Definition of the Domains of Healthy Aging

Domain	Definition
Healthy aging	Healthy aging was defined as a composite end point: surviving to the age of 70 years while being free from 11 major chronic diseases, having no impairment in memory or physical function, and being in good mental health, as defined previously for the NHS participants.
Assessment of chronic diseases	The clinical diagnoses of 11 major chronic diseases were determined from the biennial follow-up questionnaires, which were subsequently confirmed by a review of medical records or pathology reports, telephone interviews, and supplementary questionnaires. These conditions were selected because they are primary causes of mortality in the US or are considered to be highly debilitating.^26^ Previous studies have reported high validity of self-reported health information in the NHS.^27,28^ The list of 11 chronic diseases included cancer (except for nonmelanoma skin cancer), type 2 diabetes, myocardial infarction, coronary artery bypass graft surgery or percutaneous transluminal coronary angioplasty, congestive heart failure, stroke, kidney failure, chronic obstructive pulmonary disease, Parkinson disease, multiple sclerosis, and amyotrophic lateral sclerosis from the biennial follow-up questionnaires. Participants who did not report a history of any of these 11 diseases by the end of follow-up (2016) were considered to be free from chronic diseases.
Assessment of subjective memory	Subjective memory was assessed based on 7 questions included in the 2014 follow-up questionnaire regarding self-reported memory impairments about change in ability to remember things and trouble in remembering recent events, short lists, one second to the next, spoken instructions, following conversations or plot, and finding the way on familiar streets.^29,30,33^ No impairment in memory was defined as having 1 memory impairment at most.
Assessment of physical function	Physical function was assessed based on 10 questions from the Medical Outcomes Study Short-Form Health Survey, which is a 36-item questionnaire that evaluates physical function and mental health administered in 2016.^31^ The absence of impairment in physical function was defined as having no limitations in moderate activities (eg, walking a few blocks or bathing) and no more than moderate limitations on vigorous activities (eg, running, lifting heavy objects, and strenuous sports).
Assessment of mental health status	Study participants’ mental health status was assessed in 2016 by using the 15-item Geriatric Depression Scale, in which lower scores indicate better mental health.^32^ Good mental health status was defined as a Geriatric Depression Scale score of 1 or less, which corresponds to the median value in this cohort.

Abbreviation: NHS, Nurses’ Health Study.

### Assessment of Healthy Aging

Healthy aging data were collected from the 2014 and 2016 NHS questionnaires. Healthy aging was defined as surviving to the age of 70 years while being free from 11 major chronic diseases, having no impairment in memory or physical function, and being in good mental health.^33,34^ Detailed definitions for each healthy aging domain are included in Table 1. All other participants were classified as usual agers, including those who did not meet our healthy aging definition and those who died before assessing healthy aging (eFigure in Supplement 1). The baseline characteristics of participants with missing healthy aging assessments or those lost to follow-up did not differ substantially from those included in the study (eTable 1 in Supplement 1).

### Dietary Assessment

NHS participants responded to FFQs inquiring about food intake frequency expressed in standardized portions.^25^ Using the 1984 and 1986 FFQs, we calculated the intakes of each nutrient variable by multiplying the consumption frequency of each food by its nutrient content and then summing the nutrient intake for each food item. The nutrient contents were obtained from the Harvard University Food Composition Database. The carbohydrate intake variables were total carbohydrates, refined carbohydrates, high-quality carbohydrates (sum of carbohydrates from fruits, vegetables, whole grains, and legumes), carbohydrates from starchy vegetables, and dietary fiber intake, including fiber from fruits, vegetables, and cereals. Carbohydrate intake variables were expressed as a percentage of total energy intake except for the dietary fiber variables, which were expressed in calorie-adjusted grams per day. The average dietary GL for each participant was calculated by summing the products of the average carbohydrate content for each food times the number of servings consumed per day, multiplied by that food’s GI.^35^ The average dietary GI was calculated by dividing the mean GL by the total carbohydrate intake.^35^ GI values for food items in the FFQs were derived from available databases.^21,36,37^ The total carbohydrate-to-fiber ratio was calculated by dividing the intake of total carbohydrates by the intake of dietary fiber.^16,17^ Detailed definitions for each carbohydrate variable are included in eTable 2 in Supplement 1. We quantified dietary quality using the 2010 Alternative Healthy Eating Index (AHEI).^38^ The reproducibility and validity of these FFQs have been described in detail.^39,40^ The correlations between total carbohydrate and dietary fiber assessed by FFQ and dietary records were 0.64 and 0.56, respectively.^41,42^

### Assessment of Covariates

We captured covariate information from the biennial follow-up questionnaires. These included participants’ demographic, anthropometric, lifestyle (physical activity and cigarette smoking), and medical history (medication use and diagnoses of chronic conditions).

### Statistical Analysis

Our main analysis used the averaged carbohydrate intakes derived from the 1984 and 1986 FFQs. We used multivariate logistic regression to calculate the odds ratios (ORs) and 95% CIs for the association of each carbohydrate variable with healthy aging and with each healthy aging domain described previously. In the base model, we adjusted for age in 1984. In multivariate-adjusted models, we further adjusted for race, education (registered nurse, bachelor, or graduate), marital status (married or other status), postmenopausal hormone use (premenopausal, never, past user, or current user), smoking status (never smoked, former smoker, 0.1-14.9 pack-years, 15.0-29.9 pack-years; and >30 pack years), alcohol intake (0, 1.0-4.9, 5.0-14.9, and >15.0 g per day), physical activity (<3, 3.0-8.9, 9.0-17.9, 18.0-26.9, and >27 metabolic equivalents per week), mean body mass index (BMI) from 1984 and 1986 (calculated as weight in kilograms divided by height in meters squared; categorized as 22.5-24.9, 25.0-27.5, 27.6-30.0, 30.1-34.9, and >35.0), baseline history of hypertension or hypercholesterolemia (yes or no), aspirin use (never, past, or current), multivitamin use (yes or no), and total energy intake (kilocalories per day [continuous]). Race categories included White and other race (defined as Asian, Black, or any race not otherwise specified); race was included to account for potential confounding because race is associated with dietary intake and healthy aging outcomes. The final regression model was further adjusted for dietary protein intake to interpret the coefficients as the estimated association of replacing a specific percentage of energy from dietary fat with an equivalent energy percentage from each carbohydrate variable. For the trend tests in the quintile analyses, we assigned the median values of each quintile and modeled this variable continuously. We used a missing indicator for categorical covariate data; no values were missing for continuous variables.

We modeled the substitution 5% of energy from total carbohydrates or high-quality carbohydrates for the equivalent energy contribution from total protein, animal protein, plant protein, total fat, saturated fatty acids (SFA), polyunsaturated fatty acids (PUFA), trans-fatty acids (TFA), or refined carbohydrates (for high-quality carbohydrate substitution only) on the odds of healthy aging by using multivariate logistic models by simultaneously including the coefficient for total carbohydrates and the replacement macronutrient modeled continuously. The ORs and 95% CIs for the isocaloric substitution association were derived from the difference between the regression coefficients for each variable.^43^

We evaluated effect modification by baseline age, BMI, GI, dietary fiber, and AHEI by including a cross-product term between these variables and the carbohydrate intake variables, modeled continuously. We conducted subgroup analyses by fitting logistic models stratified by BMI (<25 and ≥25) or participants’ median baseline values for dietary fiber, GI, and AHEI.

In sensitivity analyses, we evaluated the association of cumulatively averaged carbohydrate intakes derived from the FFQs from 1984 to either 2002 (12- to 14-year lag) or 2006 (8- to 10-year lag) with healthy aging. For these analyses, we stopped updating dietary intakes after a diagnosis of any of the 11 chronic diseases that are part of our healthy aging definition. We also evaluated the associations for carbohydrate intake variables adjusted for dietary fiber and separately by levels of B-vitamins and polyphenols. Lastly, we analyzed GL levels cross-classified by dietary fiber levels assessed in 1984 and 1986.

Data were analyzed from January 2023 to February 2025. All statistical analyses were performed using SAS version 9.4 (SAS Institute). A 2-sided *P* < .05 was considered to be statistically significant.

## Results

### Baseline Characteristics of Participants

Of the 47 513 participants included (mean [SD] baseline age, 48.5 [6.2] years; attained age range, 70-93 years), 3706 (7.8%) met our healthy aging definition, 15 056 (31.7%) remained free from 11 chronic diseases considered, 23 196 (48.8%) did not report impairments in memory, 7300 (15.3%) had no physical function limitations, and 18 204 (38.3%) maintained good mental health. The mean baseline (SD) total carbohydrate intake was 47.0% (7.1%), including 23.2% (6.1%) refined carbohydrates and 13.6% (5.6%) high-quality carbohydrates. Other carbohydrates from lactose, fruit juice, dried fruits, and processed foods accounted for 10.2% of energy. Total carbohydrate intake was positively associated with physical activity, multivitamin use, GI, GL, and AHEI levels and inversely associated with education level, BMI, smoking status, hypertension history, aspirin use, and intakes of protein, fat, and alcohol (Table 2).

**Table 2.** Baseline Age-Adjusted Characteristics of Participants in the Nurses’ Health Study According to Quintiles of Total Carbohydrate Intake (Percentage of Total Energy)

Characteristic	Participants by quintiles of total carbohydrate intake, mean (SD)				
	1 (n = 9502)	2 (n = 9503)	3 (n = 9503)	4 (n = 9502)	5 (n = 9502)
Age, y	48.5 (6.1)	48.1 (6.2)	48.3 (6.2)	48.6 (6.3)	49.1 (6.4)
Total carbohydrates, % energy	37.0 (4.1)	43.6 (1.2)	47.1 (0.9)	50.5 (1.1)	56.6 (3.7)
Total carbohydrates, g/d	148.0 (16.3)	174.0 (5.3)	187.9 (4.7)	201.3 (5.3)	225.5 (15.3)
High-quality carbohydrates, % energy^a^	10.4 (3.7)	12.2 (4.1)	13.5 (4.4)	14.7 (5.0)	17.2 (7.2)
Refined carbohydrates, % energy,^b^	18.8 (4.7)	22.1 (4.6)	23.5 (4.9)	24.8 (5.4)	27.0 (7.1)
Carbohydrates from refined grains, % energy	9.3 (3.0)	10.6 (3.0)	11.1 (3.1)	11.2 (3.2)	11.1 (3.8)
Carbohydrates from whole grains, % energy	1.4 (1.3)	2.0 (1.6)	2.3 (1.8)	2.7 (2.1)	3.2 (2.9)
Carbohydrates from fruits (excluding fruit juice), % energy	4.6 (2.6)	5.9 (2.8)	6.6 (3.1)	7.4 (3.4)	9.1 (4.8)
Carbohydrates from vegetables (excluding potatoes and legumes), % energy	3.3 (1.3)	3.3 (1.3)	3.4 (1.3)	3.4 (1.4)	3.6 (1.7)
Carbohydrates from starchy vegetables, % energy^c^	4.1 (2.0)	4.2 (2.0)	4.2 (1.9)	4.2 (2.0)	4.2 (2.2)
Dietary fiber, g/d	13.8 (3.2)	15.6 (3.4)	16.6 (3.6)	17.6 (4)	19.3 (5.7)
Fruit fiber, g/d	2.2 (1.6)	2.9 (1.9)	3.4 (2.0)	3.8 (2.2)	4.6 (3.0)
Vegetable fiber, g/d	5.8 (2.2)	6.0 (2.2)	6.2 (2.2)	6.3 (2.3)	6.6 (3.0)
Cereal fiber, g/d	3.0 (1.4)	3.8 (1.7)	4.3 (1.9)	4.6 (2.2)	5.2 (2.9)
Ratio of total carbohydrate to fiber	11.3 (2.8)	11.9 (2.7)	12.1 (2.8)	12.4 (3.1)	13.2 (4.9)
Glycemic index	51.1 (4.1)	52 (3.4)	52.4 (3.3)	52.8 (3.4)	53.6 (3.7)
Glycemic load	76.1 (13.1)	90.6 (9.7)	98.5 (9.7)	106.1 (10.3)	120.3 (14.5)
Alternative Healthy Eating Index	44.0 (8.8)	44.1 (9.2)	44.4 (9.3)	45 (9.5)	46.9 (10.7)
Total energy, kcal/d	1644 (475)	1750 (478)	1787 (488)	1809 (489)	1800 (504)
Total protein, % of energy	19.6 (3.3)	18.8 (2.7)	18.2 (2.6)	17.6 (2.5)	16.3 (2.7)
Total fat, % of energy	38.3 (5.5)	36.2 (3.9)	34.4 (3.4)	32.5 (3.0)	28.6 (3.7)
Animal fat, % of energy	23.3 (4.9)	20.8 (3.6)	19.3 (3.2)	17.7 (3.0)	15 (3.2)
Plant fat, % of energy	15.0 (4.7)	15.4 (4.1)	15.1 (3.8)	14.8 (3.7)	13.7 (3.7)
Saturated fat, % of energy	13.9 (2.6)	13.1 (2.0)	12.4 (1.7)	11.6 (1.6)	10.2 (1.8)
Monounsaturated fat, % of energy	14.2 (2.3)	13.3 (1.7)	12.6 (1.5)	11.9 (1.4)	10.3 (1.6)
Polyunsaturated fat, % of energy	7.0 (1.8)	6.8 (1.4)	6.5 (1.3)	6.2 (1.2)	5.7 (1.2)
Trans fat, % of energy	2.2 (0.9)	2.1 (0.8)	2.1 (0.8)	2.0 (0.8)	1.8 (0.9)
Race, No. (%)					
Asian	29 (0.3)	38 (0.4)	48 (0.5)	57 (0.6)	124 (1.3)
Black	67 (0.7)	67 (0.7)	67 (0.7)	86 (0.9)	171 (1.8)
White	9084 (95.6)	9075 (95.5)	9056 (95.3)	9055 (95.3)	8827 (92.9)
Other^d^	323 (3.4)	323 (3.4)	333 (3.5)	304 (3.2)	380 (4.0)
Married, No. (%)	8105 (85.3)	8420 (88.6)	8505 (89.5)	8533 (89.8)	8371 (88.1)
Education, No. (%)					
Registered nurse	6670 (70.2)	6671 (70.2)	6776 (71.3)	6784 (71.4)	6803 (71.6)
Bachelors	1862 (19.6)	1815 (19.1)	1815 (19.1)	1843 (19.4)	1796 (18.9)
Masters or higher	969 (10.2)	1017 (10.7)	912 (9.6)	874 (9.2)	903 (9.5)
Physical activity, MET-h/wk	10.5 (17.0)	11.7 (18.3)	12.6 (19.3)	13 (18.3)	14.7 (24.8)
Alcohol intake, g/d	14.5 (16.2)	7.5 (10.0)	5.3 (7.5)	3.9 (6.1)	2.6 (4.7)
Body mass index, No. (%)^e^					
<25	5730 (60.3)	5664 (59.6)	5768 (60.7)	6053 (63.7)	6414 (67.5)
25-30	2509 (26.4)	2461 (25.9)	2471 (26.0)	2395 (25.2)	2147 (22.6)
≥30	1264 (13.3)	1378 (14.5)	1264 (13.3)	1055 (11.1)	941 (9.9)
Smoking status, No. (%)					
Never smoker	2765 (29.1)	3754 (39.5)	4286 (45.1)	4618 (48.6)	4903 (51.6)
Past smoker	3525 (37.1)	3412 (35.9)	3317 (34.9)	3126 (32.9)	2908 (30.6)
Current smoker	3212 (33.8)	2338 (24.6)	1901 (20.0)	1758 (18.5)	1691 (17.8)
Postmenopausal hormone use, No. (%)					
Premenopausal	4741 (49.9)	4742 (49.9)	4752 (50.0)	4694 (49.4)	4665 (49.1)
Never used	2737 (28.8)	2813 (29.6)	2784 (29.3)	2784 (29.3)	2746 (28.9)
Current user	1178 (12.4)	1150 (12.1)	1112 (11.7)	1150 (12.1)	1207 (12.7)
Former user	846 (8.9)	798 (8.4)	855 (9.0)	874 (9.2)	884 (9.3)
Hypertension, No. (%)	1701 (17.9)	1511 (15.9)	1482 (15.6)	1463 (15.4)	1530 (16.1)
High cholesterol, No. (%)	494 (5.2)	475 (5.0)	475 (5.0)	456 (4.8)	637 (6.7)
Multivitamin use, No. (%)	3202 (33.7)	3260 (34.3)	3431 (36.1)	3554 (37.4)	3772 (39.7)
Current aspirin use, No. (%)	6794 (71.5)	6890 (72.5)	6009 (72.7)	6841 (72.0)	6528 (68.7)

Abbreviation: MET, metabolic equivalent.

^a^ High-quality carbohydrates include carbohydrates from fruits (excluding fruit juice), nonlegume vegetables (excluding potatoes), whole grains, and legumes.

^b^ Refined carbohydrates include carbohydrates from refined grains, potatoes, and added sugars.

^c^ Carbohydrates from starchy vegetables include carbohydrates from potatoes, corn, and yams.

^d^ Other was defined as any race not otherwise specified.

^e^ Calculated as weight in kilograms divided by height in meters squared.

### Associations of Carbohydrate Intake With Healthy Aging

Over 32 years of follow-up, total carbohydrate intake (OR, 1.17; 95% CI, 1.10-1.25 per 10%-calorie increment) and high-quality carbohydrate intake (OR, 1.31; 95% CI, 1.22-1.41) were positively associated with odds of healthy aging (Table 3). Consumption of carbohydrates from whole grains, fruits, vegetables, and legumes were positively associated with odds of healthy aging (ORs ranging from 1.06; 95% CI 1.01-1.12 to 1.37; 95% CI, 1.20-1.57). In contrast, intakes of refined carbohydrates (OR, 0.87; 95% CI, 0.80-0.95) and starchy vegetables (OR, 0.90; 95% CI, 0.82-0.99) were inversely associated with odds of healthy aging (Table 3). These associations were independent of BMI (eTable 3 in Supplement 1) and were larger in magnitude for the cumulatively averaged carbohydrate intakes through either 2002 or 2006 (eTable 4 and eTable 5 in Supplement 1). Intakes of total fiber (OR, 1.17; 95% CI, 1.13-1.22) and fiber from fruits (OR, 1.14; 95% CI, 1.10-1.19), vegetables (OR, 1.11; 95% CI, 1.07-1.15), and cereals (OR, 1.07; 95% CI, 1.03-1.11) were positively associated with healthy aging odds per 1-SD increment (Table 4). We observed positive associations of GL with the odds of healthy aging. In contrast, higher GI and a higher total carbohydrate-to-fiber ratio were associated with 24% (OR, 0.76; 95% CI, 0.67-0.87) and 29% (OR, 0.71; 95% CI, 0.62-0.81) lower odds of healthy aging, respectively, comparing extreme quintiles (eTable 6 in Supplement 1). Most associations were attenuated after adjusting for dietary fiber (eTable 6 and eTable 7 in Supplement 1) but were virtually unchanged after adjusting for B-vitamins and/or polyphenol levels (eTable 8 in Supplement 1).

**Table 3.** ORs and 95% CIs of Healthy Aging Assessed in 2014 and 2016 According to Carbohydrate Intake in 1984 and 1986 Among 47 513 Participants in the Nurses’ Health Study

Carbohydrate type	Healthy aging by quintile of carbohydrate intake, OR (95% CI)					*P* value for trend^a^	Healthy aging, OR (95% CI) per given energy increment^b^
	1	2	3	4	5		
Total carbohydrates							
Healthy ager, No.	641	726	776	780	783	NA	NA
Intake, median (IQR), % energy	38.1 (35.2-40.0)	43.7 (42.6-44.6)	47.1 (46.3-47.9)	50.5 (49.6-51.5)	55.5 (53.9-58.3)	NA	NA
Age-adjusted model	1.00 [Reference]	1.06 (0.95-1.19)	1.18 (1.05-1.32)	1.24 (1.11-1.38)	1.34 (1.20-1.50)	<.001	1.18 (1.13-1.25)
Multivariate model 1^c^	1.00 [Reference]	1.00 (0.89-1.13)	1.05 (0.93-1.19)	1.07 (0.94-1.21)	1.13 (0.99-1.28)	.04	1.09 (1.03-1.15)
Multivariate model 2^d^	1.00 [Reference]	1.04 (0.92-1.17)	1.12 (0.98-1.26)	1.16 (1.02-1.32)	1.29 (1.12-1.48)	<.001	1.17 (1.10-1.25)
High-quality carbohydrates^e^							
Healthy ager, No.	679	762	704	804	757	NA	NA
Intake, median (IQR), % energy	7.3 (6.1-8.2)	10.3 (9.7-11.0)	12.8 (12.2-13.5)	15.7 (14.9-16.7)	20.8 (19.0-23.8)	NA	NA
Age-adjusted model	1.00 [Reference]	1.29 (1.15-1.44)	1.32 (1.17-1.47)	1.80 (1.61-2.01)	2.21 (1.97-2.48)	<.001	1.64 (1.54-1.74)
Multivariate model 1^c^	1.00 [Reference]	1.14 (1.02-1.28)	1.08 (0.96-1.22)	1.40 (1.25-1.58)	1.54 (1.36-1.74)	<.001	1.32 (1.23-1.42)
Multivariate model 2^d^	1.00 [Reference]	1.13 (1.01-1.27)	1.07 (0.95-1.21)	1.38 (123-1.56)	1.51 (1.33-1.71)	<.001	1.31 (1.22-1.41)
Refined carbohydrates^f^							
Healthy ager, No.	610	695	788	842	771	NA	NA
Intake, median (IQR), % energy	15.8 (13.8-17.2)	20.0 (19.2-20.8)	23.0 (22.3-23.7)	26.0 (25.1-26.9)	30.8 (29.1-33.5)	NA	NA
Age-adjusted model	1.00 [Reference]	0.94 (0.83-1.05)	0.93 (0.83-1.05)	0.90 (0.81-1.01)	0.72 (0.64-0.81)	<.001	0.81 (0.77-0.86)
Multivariate model 1^c^	1.00 [Reference]	0.95 (0.84-1.07)	0.97 (0.86-1.09)	0.94 (0.83-1.06)	0.80 (0.71-0.91)	.001	0.86 (0.81-0.92)
Multivariate model 2^d^	1.00 [Reference]	0.96 (0.84-1.08)	0.98 (0.87-1.12)	0.97 (0.85-1.11)	0.85 (0.73-0.98)	.04	0.87 (0.80-0.95)
Carbohydrates from whole grains							
Healthy ager, No.	606	733	770	797	800	NA	NA
Intake, median (IQR), % energy	0.5 (0.3-0.6)	1.0 (0.9-1.2)	1.7 (1.5-1.9)	2.7 (2.4-3.0)	4.9 (4.1-6.4)	NA	NA
Age-adjusted model	1.00 [Reference]	1.15 (1.03-1.29)	1.23 (1.10-1.38)	1.34 (1.20-1.50)	1.65 (1.48-1.85)	<.001	1.41 (1.31-1.53)
Multivariate model 1^c^	1.00 [Reference]	1.11 (0.99-1.26)	1.10 (0.98-1.24)	1.11 (0.99-1.25)	1.22 (1.08-1.37)	.005	1.11 (1.02-1.21)
Multivariate model 2^d^	1.00 [Reference]	1.11 (0.99-1.25)	1.10 (0.97-1.23)	1.10 (0.98-1.25)	1.20 (1.07-1.36)	.008	1.11 (1.01-1.21)
Carbohydrates from fruits excluding fruit juice							
Healthy ager, No.	699	764	746	761	736	NA	NA
Intake, median (IQR), % energy	2.6 (1.9-3.2)	4.5 (4.1-4.9)	6.2 (5.7-6.6)	8.1 (7.5-8.7)	11.6 (10.4-13.7)	NA	NA
Age-adjusted model	1.00 [Reference]	1.24 (1.11-1.39)	1.34 (1.20-1.50)	1.67 (1.49-1.86)	2.07 (1.85-2.32)	<.001	1.39 (1.33-1.46)
Multivariate model 1^c^	1.00 [Reference]	1.08 (0.97-1.22)	1.10 (0.98-1.23)	1.31 (1.16-1.47)	1.50 (1.33-1.69)	<.001	1.22 (1.16-1.28)
Multivariate model 2^d^	1.00 [Reference]	1.08 (0.96-1.21)	1.09 (0.97-1.22)	1.30 (1.15-1.46)	1.48 (1.31-1.68)	<.001	1.22 (1.15-1.28)
Carbohydrates from vegetables excluding potatoes and legumes							
Healthy ager, No.	695	815	710	736	750	NA	NA
Intake, median (IQR), % energy	1.9 (1.6-2.1)	2.6 (2.4-2.7)	3.2 (3.0-3.3)	3.9 (3.7-4.1)	5.1 (4.7-5.9)	NA	NA
Age-adjusted model	1.00 [Reference]	1.28 (1.15-1.43)	1.20 (1.07-1.34)	1.31 (1.17-1.47)	1.54 (1.38-1.73)	<.001	1.63 (1.45-1.84)
Multivariate model 1^c^	1.00 [Reference]	1.19 (1.06-1.33)	1.10 (0.98-1.24)	1.19 (1.06-1.34)	1.33 (1.18-1.50)	<.001	1.41 (1.24-1.61)
Multivariate model 2^d^	1.00 [Reference]	1.18 (1.05-1.32)	1.09 (0.97-1.22)	1.17 (1.04-1.31)	1.30 (1.15-1.46)	<.001	1.37 (1.20-1.57)
Carbohydrates from starchy vegetables^g^							
Healthy ager, No.	739	770	739	742	716	NA	NA
Intake, median (IQR), % energy	2.0 (1.6-2.2)	3.0 (2.7-3.2)	3.9 (3.7-4.1)	5.0 (4.7-5.3)	6.7 (6.1-7.8)	NA	NA
Age-adjusted model	1.00 [Reference]	0.86 (0.77-0.96)	0.77 (0.69-0.86)	0.77 (0.69-0.87)	0.75 (0.67-0.84)	<.001	0.78 (0.71-0.86)
Multivariate model 1^c^	1.00 [Reference]	0.92 (0.82-1.03)	0.85 (0.76-0.96)	0.88 (0.79-0.99)	0.88 (0.78-0.99)	.05	0.90 (0.82-0.99)
Multivariate model 2^d^	1.00 [Reference]	0.93 (0.83-1.04)	0.86 (0.76-0.96)	0.89 (0.79-1.00)	0.89 (0.79-1.01)	.08	0.90 (0.82-0.99)
Carbohydrates from legumes							
Healthy ager, No.	735	750	730	724	767	NA	NA
Intake, median (IQR), % energy	0.4 (0.3-0.5)	0.8 (0.7-0.8)	1.0 (1.0-1.1)	1.4 (1.3-1.5)	2.0 (1.7-2.4)	NA	NA
Age-adjusted model	1.00 [Reference]	1.09 (0.98-1.22)	1.12 (1.00-1.25)	1.14 (1.02-1.27)	1.33 (1.19-1.49)	<.001	1.12 (1.07-1.17)
Multivariate model 1^c^	1.00 [Reference]	1.08 (0.96-1.21)	1.08 (0.96-1.21)	1.09 (0.97-1.22)	1.21 (1.08-1.36)	.001	1.07 (1.02-1.12)
Multivariate model 2^d^	1.00 [Reference]	1.08 (0.96-1.21)	1.08 (0.96-1.21)	1.08 (0.96-1.21)	1.20 (1.07-1.35)	.003	1.06 (1.01-1.12)

Abbreviations: NA, not applicable; OR, odds ratio.

^a^ *P* for trend was calculated by assigning median values to each quintile and was treated as a continuous variable.

^b^ Per 10% increment for total carbohydrates, high-quality carbohydrates, and refined carbohydrates; per 5% increment for carbohydrates from whole grains, carbohydrates from fruits (excluding fruit juice), carbohydrates from vegetables (excluding potatoes and legumes), and carbohydrates from starchy vegetables; and per 1% increase for carbohydrates from legumes. ORs greater than 1 denote higher odds of healthy aging.

^c^ Multivariate model 1 was adjusted for baseline age (continuous), race (White and other race [ie, Asian, Black, and any race not otherwise specified]), education (registered nurse, bachelor, or graduate), marital status (married or other), postmenopausal hormone use (premenopausal, never, past user, or current user), smoking status (never smoked; former smoker; and 0.1-14.9, 15.0-29.9, and >30 pack-years), alcohol intake (0, 0.1-4.9, 5.0-14.9, and >15.0 g/d), physical activity (<3, 3-8.9, 9-17.9, 18-26.9, and >27 metabolic equivalent tasks/wk), baseline history of hypertension or hypercholesterolemia (yes or no), aspirin use (never, past, or current), multivitamin use (yes or no), total energy intake (kilocalories/day, quintiles), and 1984 and 1986 mean body mass index (calculated as weight in kilograms divided by height in meters squared; categorized as <22.5, 22.5-24.9, 25.0-27.5, 27.5-30.0, 30.0-34.9, and >35.0).

^d^ Multivariate model 2 included covariates in multivariate model 1 with additional adjustment for dietary protein.

^e^ High-quality carbohydrates include carbohydrates from fruits (excluding fruit juice), nonlegume vegetables (excluding potatoes), whole grains, and legumes.

^f^ Refined carbohydrates include carbohydrates from refined grains, potatoes, and added sugars.

^g^ Carbohydrates from starchy vegetables includes carbohydrates from potatoes, corn, and yams.

**Table 4.** ORs and 95% CIs of Healthy Aging Assessed in 2014 and 2016 According to Dietary Fiber Intake in 1984 and 1986 Among 47 513 Participants in the Nurses’ Health Study

Fiber type	Healthy aging by quintile of fiber intake, OR (95% CI)					*P* value for trend^a^	Healthy aging, OR (95% CI) per 1-SD increment^b^
	1	2	3	4	5		
Total fiber							
Healthy ager, No.	659	762	718	781	786	NA	NA
Intake, median (IQR), g	11.5 (10.3-12.3)	14.1 (13.6-14.6)	16.0 (15.5-16.5)	18.3 (17.7-19.1)	22.3 (20.9-24.6)	NA	NA
Age-adjusted model	1.00 [Reference]	1.26 (1.13-1.41)	1.31 (1.17-1.47)	1.61 (1.44-1.80)	2.12 (1.90-2.37)	<.001	1.30 (1.25-1.34)
Multivariate model 1^c^	1.00 [Reference]	1.16 (1.04-1.31)	1.13 (1.00-1.27)	1.34 (1.19-1.51)	1.58 (1.40-1.78)	<.001	1.18 (1.13-1.22)
Multivariate model 2^d^	1.00 [Reference]	1.16 (1.03-1.30)	1.12 (1.00-1.26)	1.33 (1.18-1.50)	1.55 (1.37-1.76)	<.001	1.17 (1.13-1.22)
Fruit fiber							
Healthy ager, No.	628	779	786	750	763	NA	NA
Intake, median (IQR), g	1.0 (0.7-1.2)	1.9 (1.7-2.1)	2.4 (2.6-3.1)	4.1 (3.8-4.5)	6.3 (5.5-7.7)	NA	NA
Age-adjusted model	1.00 [Reference]	1.32 (1.18-1.47)	1.44 (1.29-1.61)	1.66 (1.48-1.86)	2.05 (1.83-2.30)	<.001	1.23 (1.18-1.27)
Multivariate model 1^c^	1.00 [Reference]	1.20 (1.07-1.35)	1.23 (1.09-1.39)	1.39 (1.23-1.56)	1.63 (1.44-1.84)	<.001	1.14 (1.10-1.19)
Multivariate model 2^d^	1.00 [Reference]	1.20 (1.07-1.35)	1.23 (1.09-1.38)	1.37 (1.22-1.55)	1.61 (1.42-1.82)	<.001	1.14 (1.10-1.19)
Vegetable fiber							
Healthy ager, No.	687	753	766	695	805	NA	NA
Intake, median (IQR), g	3.6 (3.1-4.0)	4.8 (4.6-5.1)	5.8 (5.6-6.1)	7.0 (6.7-7.4)	9.2 (8.4-10.6)	NA	NA
Age-adjusted model	1.00 [Reference]	1.20 (1.07-1.34)	1.31 (1.17-1.46)	1.23 (1.10-1.38)	1.65 (1.48-1.85)	<.001	1.16 (1.12-1.20)
Multivariate model 1^c^	1.00 [Reference]	1.12 (1.00-1.25)	1.23 (1.09-1.38)	1.10 (0.98-1.24)	1.47 (1.31-1.65)	<.001	1.12 (1.08-1.16)
Multivariate model 2^d^	1.00 [Reference]	1.11 (0.99-1.25)	1.21 (1.08-1.36)	1.09 (0.97-1.23)	1.44 (1.28-1.62)	<.001	1.11 (1.07-1.15)
Cereal fiber							
Healthy ager, No.	590	745	767	810	794	NA	NA
Intake, median (IQR), g	2.1 (1.8-2.4)	3.0 (2.8-3.2)	3.7 (3.5-3.9)	4.7 (4.4-5.0)	6.8 (6.0-8.2)	NA	NA
Age-adjusted model	1.00 [Reference]	1.16 (1.03-1.30)	1.24 (1.11-1.39)	1.46 (1.30-1.63)	1.66 (1.48-1.86)	<.001	1.17 (1.14-1.21)
Multivariate model 1^c^	1.00 [Reference]	1.08 (0.96-1.22)	1.09 (0.96-1.23)	1.19 (1.06-1.35)	1.21 (1.07-1.36)	.001	1.07 (1.03-1.11)
Multivariate model 2^d^	1.00 [Reference]	1.09 (0.96-1.23)	1.10 (0.97-1.24)	1.21 (1.07-1.37)	1.22 (1.08-1.37)	.001	1.07 (1.03-1.11)

Abbreviations: NA, not applicable; OR, odds ratio.

^a^ *P* for trend was calculated by assigning median values to each quintile and was treated as a continuous variable.

^b^ Fiber intake variable SDs were: total fiber, 4.5 g/day; fruit fiber, 2.3 g/day; vegetable fiber, 2.4 g/day; and cereal fiber, 2.2 g/day.

^c^ Multivariate model 1 was adjusted for baseline age (continuous), race (White and other race [ie, Asian, Black, and any race not otherwise specified]), education (registered nurse, bachelor, or graduate), marital status (married or other), postmenopausal hormone use (premenopausal, never, past user, or current user), smoking status (never smoked; former smoker; and 0.1-14.9, 15.0-29.9, and >30 pack-years), alcohol intake (0, 0.1-4.9, 5.0-14.9, and >15.0 g/d), physical activity (<3, 3-8.9, 9-17.9, 18-26.9, and >27 metabolic equivalent tasks/wk), baseline history of hypertension or hypercholesterolemia (yes or no), aspirin use (never, past, or current), multivitamin use (yes or no), total energy intake (kilocalories/day, quintiles), and mean 1984 and 1986 body mass index (calculated as weight in kilograms divided by height in meters squared; categorized as <22.5, 22.5-24.9, 25.0-27.5, 27.5-30.0, 30.0-34.9, and >35.0).

^d^ Multivariate model 2 included covariates in multivariate model 1 with additional adjustment for dietary protein.

The associations of carbohydrate intake with each domain of healthy aging are presented in eTable 9 in Supplement 1. Total carbohydrate consumption was associated with 4% (OR, 1.04; 95% CI, 1.01-1.08) to 8% (OR, 1.08; 95% CI, 1.03-1.14) higher odds of all domains except for the absence of chronic diseases. High-quality carbohydrate intake was associated with 8% (OR, 1.08; 95% CI 1.04-1.12) to 24% (OR, 1.24; 95% CI, 1.17-1.31) higher odds of all domains. Conversely, refined carbohydrate intake was associated with 6% (OR, 0.94; 95% CI, 0.90-0.99) to 9% (OR, 0.91; 95% CI, 0.85-0.97) lower odds of all healthy aging domains except for lack of memory impairments. Intake of carbohydrates from fruits was associated with 6% (OR, 1.06; 95% CI, 1.03-1.10) to 16% (OR, 1.16; 95% CI, 1.11-1.21) higher odds of all 4 domains. Consumption of carbohydrates from vegetables was associated with 11% (OR, 1.11; 95% CI, 1.02-1.20) to 31% (OR, 1.31; 95% CI, 1.18-1.46) higher odds of all domains except for the absence of chronic diseases, and whole grain carbohydrate intake was associated with 7% (OR, 1.07; 95% CI, 1.02-1.12) to 10% (1.10; 95% CI, 1.03-1.18) higher odds of all domains except for lack of memory impairments. Intake of carbohydrates from starchy vegetables was associated with 8% lower odds of absence of chronic diseases (OR, 0.92; 95% CI, 0.88-0.97).

Intakes of total and fruit fiber were associated with 5% (OR, 1.05; 95% CI, 1.03-1.07) to 15% (OR, 1.15; 95% CI, 1.11-1.18) higher odds of all healthy aging domains. Cereal fiber consumption was associated with higher odds of all domains, except for lack of memory impairments. Vegetable fiber intake was associated with higher odds of all domains except for the absence of chronic diseases (eTable 10 in Supplement 1).

GL was associated with higher odds of good mental health but not with other domains. In contrast, higher GI and a higher total carbohydrate-to-fiber ratio were associated with lower odds of all domains (eTable 11 in Supplement 1).

Substituting 5% of energy from total carbohydrates for total protein, animal or plant protein, or PUFA was associated with 7% (OR 0.93; 95% CI, 0.88-0.99) to 37% (0.63; 95% CI, 0.51-0.78) lower odds of healthy aging. Conversely, substituting total carbohydrates for total fat or TFA was associated with 7% (OR, 1.07; 95% CI, 1.03-1.11) and 9% (1.09; 95% CI, 1.02-1.15) higher odds of healthy aging, respectively (Figure and eTable 12 in Supplement 1). Substituting high-quality carbohydrates for refined carbohydrates, animal protein, total fat, or TFA was associated with 8% (OR, 1.08; 95% CI, 1.01-1.16) to 16% (OR, 1.16; 95% CI, 1.11-1.21) higher odds of healthy aging; no associations were observed for total or plant protein, SFA, or PUFA replacements.

**Figure.**
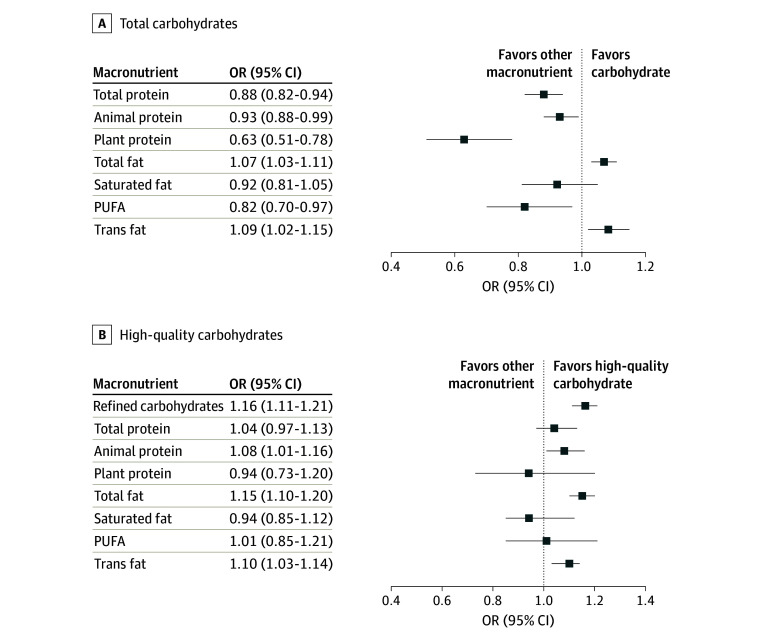
Odds Ratios (ORs) and 95% CIs for Healthy Aging Associated With the Isocaloric Substitution of Carbohydrates for Other Macronutrients Among 47 513 Participants in the Nurses’ Health Study The isocaloric substitutions can be interpreted as the effect of increasing the calories contributed by either total carbohydrates (A) or high-quality carbohydrates (B) by 5% total energy per day while decreasing the corresponding calories contributed by dietary protein, dietary fat, or other dietary carbohydrate variables on the odds of healthy aging. The OR for trans fat replacement is expressed in a 1% calorie per day increment. High-quality carbohydrates include carbohydrates from fruits (excluding fruit juice), nonlegume vegetables (excluding potatoes), whole grains, and legumes. Refined carbohydrates include carbohydrates from refined grains, potatoes, and added sugars. PUFA indicates polyunsaturated fatty acids.

In our stratified analyses, we observed larger-magnitude associations of total carbohydrates and GL with healthy aging among participants with dietary fiber consumption above the median level for the cohort and for participants with GI below the median (eTable 13 in Supplement 1). Lastly, we observed larger positive associations for participants with simultaneous GL and dietary fiber intakes above the median levels than those below the median (eTable 14 in Supplement 1).

## Discussion

In our cohort study of women with attained ages of 70 to 93 years, we observed positive (favorable) associations of intakes of total and high-quality carbohydrates during midlife with the odds of healthy aging, including favorable associations of intakes of carbohydrates from whole grains, fruits, vegetables, and legumes; total dietary fiber; and fiber from fruits, vegetables, and cereals, with the odds of healthy aging and several domains of healthy aging. Conversely, intakes of refined carbohydrates and carbohydrates from starchy vegetables were unfavorably associated with healthy aging. We observed larger-magnitude associations for carbohydrate intakes cumulatively averaged over 6 to 7 FFQs through 2002 or 2006, supporting the robustness of our findings. These associations were not modified by age, BMI, or dietary quality; however, total carbohydrate and GL had larger-magnitude associations with healthy aging among participants with dietary fiber intakes above the cohort’s median level and GI levels below the median. In substitution analyses, high-quality carbohydrate intake was associated with higher odds of healthy aging when replacing energy from refined carbohydrates, total fat, animal protein, and TFA, but not when compared with energy from total or plant protein, SFA, or PUFA.

Our results are consistent with a study among adults in Australia observing that dietary fiber, including cereal and fruit fiber, was positively associated with healthy aging.^15^ However, that study did not observe associations for total carbohydrate intake, GI, or GL.^15^ A separate cross-sectional study in 2 cohorts in Greece did not observe associations of carbohydrate intake—modeled jointly with protein intake—with odds of successful aging^44^ except for favorable associations for whole grain consumption.^45^

Our findings for carbohydrate-specific sources are consistent with prior evidence for chronic disease incidence and mortality.^12,13,14^ Similarly, dietary fiber intake has been associated with reduced risks of total mortality and chronic disease risk,^46^ increased life span^47^ and telomere length,^48^ lower prevalence of depressive symptoms,^49^ and favorable memory scores.^50^ Additionally, fiber intake has been favorably associated with physical^51,52,53,54,55,56,57^ and cognitive^58,59^ function in older adulthood. Conversely, refined carbohydrate intake was associated with lower cognition scores.^50^

There are several possible mechanisms explaining our findings. In our study, higher total carbohydrate intake corresponded to higher intakes of dietary fiber and high-quality carbohydrates, which may explain their favorable associations with healthy aging; this is supported by significantly larger positive associations observed for participants in the stratum of higher fiber intake and the attenuation of our associations with healthy aging after adjusting for dietary fiber intake. The unfavorable associations of GI with healthy aging are consistent with observational evidence for GI’s role in increasing chronic disease risk and mortality^20,60,61^ and positive associations of GI with odds of depressive symptoms.^49,62,63^ However, our findings for GL were unexpected, given that higher GL has been associated with increased cardiometabolic disease risk^20^ and lower cognitive scores.^64,65^ Of note, our positive GL associations were attenuated after adjusting for dietary fiber intake and, in stratified analyses, were larger among participants with higher fiber intake, suggesting that dietary GL partially captured the association of fiber-containing foods and that the interpretation of our GL and total carbohydrate findings may depend on fiber intake.

The beneficial role of dietary fiber in cardiovascular disease and all-cause mortality^66,67^ may be mediated by gut microbiome metabolites (most notably, indolepropionate).^68,69,70^ However, the mechanisms linking fiber intake to healthy longevity remain unknown. Our findings suggest that the associations of fiber intake with the individual healthy aging domains may differ by food source, consistent with prior evidence.^15,66^ Dietary fiber is associated with decreased levels of chronic inflammation markers, which are implicated in metabolic pathways related to aging.^71,72^ We note that B-vitamins and polyphenols in fiber-rich foods may have contributed to our observed associations; however, our results were not materially attenuated after adjusting for these variables.^33,73,74^

### Strengths and Limitations

Our study strengths include the assessment of carbohydrate intake in midlife, which represents an important etiologic window for the onset of chronic diseases and physical and cognitive function declines. The long time-lag between dietary assessment and the evaluation of health status minimized the possibility of reverse causation biasing our results. Further strengths include the evaluation of multiple domains of aging, a large sample size with high follow-up rates, and adjustment for several covariates to minimize the likelihood of residual confounding.

Among our limitations, the study population included mostly well-educated participants who consumed a higher proportion of high-quality carbohydrates than the general US population, which may limit the generalizability of our findings to populations with different characteristics. While measurement error in dietary exposure assessment is inevitable, the FFQ has had good validity when compared with dietary records and biomarkers of intake. We excluded participants with chronic diseases at baseline to minimize reverse causation bias; however, we could not exclude those with physical function or memory function limitations because those data were unavailable for the baseline questionnaires. Our diet and outcome data are more than 10 years old; therefore, the effect of more recent changes in diet or detection of outcomes cannot be addressed. Additionally, we lacked time-to-event data for most domains to conduct survival analyses. Further, we cannot exclude the possibility of residual or unmeasured confounding explaining our results; however, we controlled for several determinants of healthy aging.

## Conclusions

In this cohort study of women, intakes of carbohydrates from fruits, vegetables, whole grains, legumes, and dietary fiber in midlife were favorably associated with healthy aging. Conversely, intakes of refined carbohydrates were unfavorably associated with healthy aging. Furthermore, intakes of total dietary fiber and individual fiber sources were favorably associated with several domains of health status. Future research needs to verify these findings in other cohorts and to elucidate potential mechanisms.

## Notes

Supplement 1. **eFigure.** Participant Flow Chart **eTable 1.** Baseline Age-Adjusted Characteristics of Participants in the Nurses’ Health Study **eTable 2.** Primary Contributors for Each Carbohydrate Variable **eTable 3.** ORs (95% CIs) of Individual Domains of Healthy Aging According to Intake of Carbohydrates In 1984/1986 Among 47,513 Participants in the Nurses’ Health Study Without Adjustment for BMI **eTable 4.** ORs (95% CIs) of Healthy Aging (n=3,706) According to Intake of Carbohydrates Cumulatively Averaged Through 2002 or 2006 Among 47,513 Participants in the Nurses’ Health Study **eTable 5.** ORs (95% CIs) of Individual Domains of Healthy Aging According to Intake of Carbohydrates Cumulatively Averaged Through 2002 or 2006 Among 47,513 Participants in the Nurses’ Health Study **eTable 6.** ORs (95% CIs) of Healthy Aging (n = 3,706) Assessed in 2014/2016 According to Measures of Carbohydrate Quality in 1984/1986 Among 47,513 Participants in the Nurses’ Health Study **eTable 7.** ORs (95% CIs) of Individual Domains of Healthy Aging According to Intake of Carbohydrates in 1984/1986 Among 47,513 Participants In the Nurses’ Health Study With Further Adjustment for Dietary Fiber **eTable 8.** ORs (95% CIs) of Individual Domains of Healthy Aging According to Intake of Carbohydrates in 1984/1986 Among 47,513 Participants in the Nurses’ Health Study With Additional Adjustment for B-Vitamins and Polyphenols **eTable 9.** ORs (95% CIs) of Individual Domains of Healthy Aging According to Intake of Carbohydrate in 1984/1986 Among 47,513 Participants in the Nurses’ Health Study **eTable 10.** ORs (95% CIs) of Individual Domains of Healthy Aging According to Intake of Dietary Fiber in 1984/1986 Among 47,513 Participants in the Nurses’ Health Study **eTable 11.** ORs (95% CIs) of Individual Domains of Healthy Aging According to Indices of Carbohydrate Quality in 1984/1986 Among 47,513 Participants in the Nurses’ Health Study **eTable 12.** ORs (95% CIs) of Healthy Aging Associated With Isocaloric Substitution of Carbohydrate (Total, High-Quality, or From Whole Grains) for Other Macronutrients Modeled in 5%-Energy Increments in 47,513 Participants in the Nurses’ Health Study **eTable 13.** ORs (95% CIs) of Healthy Aging According to Baseline (1984/1986) Total Carbohydrate Intake and Glycemic Load Stratified by GI and AHEI Among 47,513 Participants in the Nurses’ Health Study **eTable 14.** ORs (95% CIs) of Healthy Aging (n = 3,706) Assessed in 2014/2016 According to the Dietary Glycemic Load Cross-Classified by Total Dietary Fiber Levels Assessed in 1984/1986 Among 47,513 Participants in the Nurses’ Health Study

Supplement 2.
Data Sharing Statement
