# Supplementary material for: Dietary Carbohydrate Intake, Carbohydrate Quality, and Healthy Aging in Women
Source: JAMA Netw Open. 2025 May 16;8(5):e2511056. doi: 10.1001/jamanetworkopen.2025.11056 (PMC12084844; doi:10.1001/jamanetworkopen.2025.11056)
Supplement: Supplement 1. — eFigure. Participant Flow Chart eTable 1. Baseline Age-Adjusted Characteristics of Participants in the Nurses’ Health Study eTable 2. Primary Contributors for Each Carbohydrate Variable eTable 3. ORs (95% CIs) of Individual Domains of Healthy Aging According to Intake of Carbohydrates In 1984/1986 Among 47,513 Participants in the Nurses’ Health Study Without Adjustment for BMI eTable 4. ORs (95% CIs) of Healthy Aging (n=3,706) According to Intake of Carbohydrates Cumulatively Averaged Through 2002 or 2006 Among 47,513 Participants in the Nurses’ Health Study eTable 5. ORs (95% CIs) of Individual Domains of Healthy Aging According to Intake of Carbohydrates Cumulatively Averaged Through 2002 or 2006 Among 47,513 Participants in the Nurses’ Health Study eTable 6. ORs (95% CIs) of Healthy Aging (n = 3,706) Assessed in 2014/2016 According to Measures of Carbohydrate Quality in 1984/1986 Among 47,513 Participants in the Nurses’ Health Study eTable 7. ORs (95% CIs) of Individual Domains of Healthy Aging According to Intake of Carbohydrates in 1984/1986 Among 47,513 Participants In the Nurses’ Health Study With Further Adjustment for Dietary Fiber eTable 8. ORs (95% CIs) of Individual Domains of Healthy Aging According to Intake of Carbohydrates in 1984/1986 Among 47,513 Participants in the Nurses’ Health Study With Additional Adjustment for B-Vitamins and Polyphenols eTable 9. ORs (95% CIs) of Individual Domains of Healthy Aging According to Intake of Carbohydrate in 1984/1986 Among 47,513 Participants in the Nurses’ Health Study eTable 10. ORs (95% CIs) of Individual Domains of Healthy Aging According to Intake of Dietary Fiber in 1984/1986 Among 47,513 Participants in the Nurses’ Health Study eTable 11. ORs (95% CIs) of Individual Domains of Healthy Aging According to Indices of Carbohydrate Quality in 1984/1986 Among 47,513 Participants in the Nurses’ Health Study eTable 12. ORs (95% CIs) of Healthy Aging Associated With Isocaloric Substitution of Carbohydrate (Total, [file jamanetwopen-e2511056-s001.pdf]

Supplemental Online Content

Ardisson Korat AV, Duscova E, Shea MK, et al. Dietary carbohydrate intake, carbohydrate quality, and healthy aging in women. *JAMA Netw. Open*. 2025;8(5):e2511056. doi:10.1001/jamanetworkopen.2025.11056

eFigure. Participant Flow Chart

eTable 1. Baseline Age-Adjusted Characteristics of Participants in the Nurses' Health Study

eTable 2. Primary Contributors for Each Carbohydrate Variable

eTable 3. ORs (95% CIs) of Individual Domains of Healthy Aging According to Intake of Carbohydrates In 1984/1986 Among 47,513 Participants in the Nurses' Health Study Without Adjustment for BMI

eTable 4. ORs (95% CIs) of Healthy Aging (n=3,706) According to Intake of Carbohydrates Cumulatively Averaged Through 2002 or 2006 Among 47,513 Participants in the Nurses' Health Study

eTable 5. ORs (95% CIs) of Individual Domains of Healthy Aging According to Intake of Carbohydrates Cumulatively Averaged Through 2002 or 2006 Among 47,513 Participants in the Nurses' Health Study

eTable 6. ORs (95% CIs) of Healthy Aging (n = 3,706) Assessed in 2014/2016 According to Measures of Carbohydrate Quality in 1984/1986 Among 47,513 Participants in the Nurses' Health Study

eTable 7. ORs (95% CIs) of Individual Domains of Healthy Aging According to Intake of Carbohydrates in 1984/1986 Among 47,513 Participants In the Nurses' Health Study With Further Adjustment for Dietary Fiber

eTable 8. ORs (95% CIs) of Individual Domains of Healthy Aging According to Intake of Carbohydrates in 1984/1986 Among 47,513 Participants in the Nurses' Health Study With Additional Adjustment for B-Vitamins and Polyphenols

eTable 9. ORs (95% CIs) of Individual Domains of Healthy Aging According to Intake of Carbohydrate in 1984/1986 Among 47,513 Participants in the Nurses' Health Study

eTable 10. ORs (95% CIs) of Individual Domains of Healthy Aging According to Intake of Dietary Fiber in 1984/1986 Among 47,513 Participants in the Nurses' Health Study

eTable 11. ORs (95% CIs) of Individual Domains of Healthy Aging According to Indices of Carbohydrate Quality in 1984/1986 Among 47,513 Participants in the Nurses' Health Study

© 2025 Ardisson Korat et al. *JAMA Network Open*.

eTable 12. ORs (95% CIs) of Healthy Aging Associated With Isocaloric Substitution of Carbohydrate (Total, High-Quality, or From Whole Grains) for Other Macronutrients Modeled in 5%-Energy Increments in 47,513 Participants in the Nurses' Health Study

eTable 13. ORs (95% CIs) of Healthy Aging According to Baseline (1984/1986) Total Carbohydrate Intake and Glycemic Load Stratified by GI and AHEI Among 47,513 Participants in the Nurses' Health Study

eTable 14. ORs (95% CIs) of Healthy Aging (n = 3,706) Assessed in 2014/2016 According to the Dietary Glycemic Load Cross-Classified by Total Dietary Fiber Levels Assessed in 1984/1986 Among 47,513 Participants in the Nurses' Health Study

This supplemental material has been provided by the authors to give readers additional information about their work.

© 2025 Ardisson Korat et al. *JAMA Network Open*.

eFigure. Participant Flow Chart

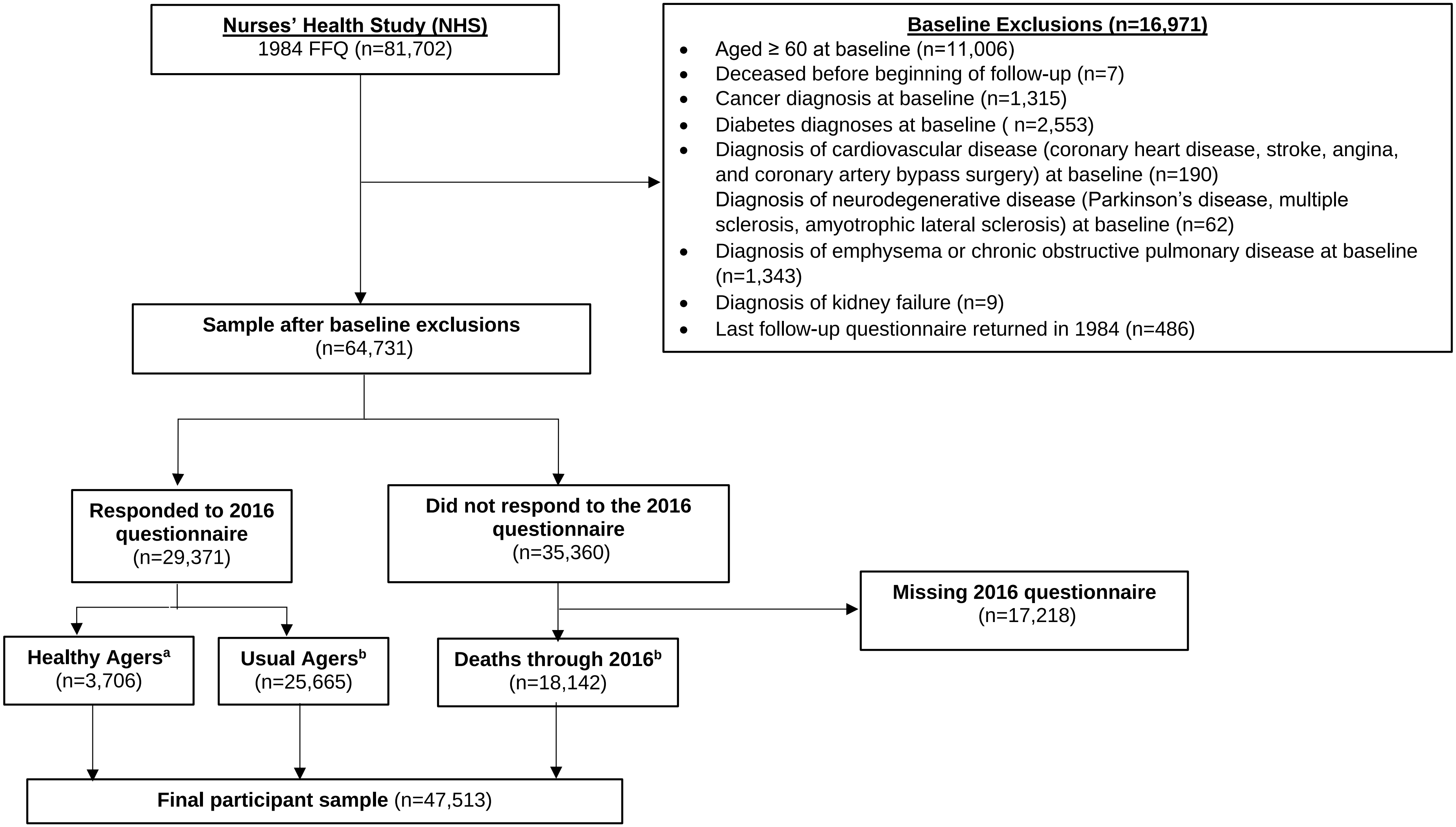

a. Healthy agers met our healthy aging definition: being free from 11 major chronic diseases, having no impairment in memory or physical function, and being in good mental health.
b. Usual al agers did not meet the definition of healthy aging for one or more domains (having a diagnosis of 1 of 11 chronic diseases, having impairments in memory or physical function of mental health), Participants who died before 2016 were also considered usual agers in our study.

© 2025 Ardisson Korat et al. *JAMA Network Open*.

eTable 1. Baseline Age-Adjusted Characteristics of Participants in the Nurses' Health Study

	Participants in the present study (n=47,513)	Participants with valid dietary and healthy aging assessments (n=61,264)	Excluded participants (n=34,179)	Participants with valid 1984 and 1986 FFQ (n=81,692)
Age, years[a]	48.5 (6.2)	50.6 (7.3)	55.3 (6.1)	50.5 (7.2)
Total carbohydrates, % energy[b]	47.0 (7.1)	47.2 (7.2)	47.2 (7.1)	47.1 (7.2)
High-quality carbohydrates, % energy[b,c]	13.6 (5.6)	14.2 (5.6)	14.0 (5.9)	13.8 (6.2)
Refined carbohydrates, % energy[b,d]	23.2 (6.1)	22.9 (6.1)	22.9 (6.0)	23.0 (6.1)
Carbohydrates from refined grains, % energy[b]	11.1 (3.0)	10.5 (3.3)	10.3 (3.5)	10.5 (3.4)
Carbohydrates from whole grains, % energy[b]	2.3 (2.1)	2.4 (2.2)	2.5 (2.3)	2.4 (2.2)
Carbohydrates from fruits (excluding fruit juice), % energy[b]	6.8 (3.8)	7.3 (3.9)	7.2 (4.0)	7.1 (3.9)
Carbohydrates from vegetables (excluding potatoes and legumes), % energy[b]	3.4 (1.4)	3.4 (1.4)	3.8 (1.5)	3.5 (1.5)
Carbohydrates from starchy vegetables, % energy[b,e]	4.2 (2.0)	4.2 (2.0)	4.2 (2.1)	4.2 (2.3)
Dietary fiber, g/day[b]	16.6 (4.5)	16.9 (4.6)	16.6 (4.5)	16.7 (4.7)
Fruit fiber, g/day[b]	3.4 (2.3)	3.5 (2.4)	3.6 (2.5)	3.6 (2.4)
Vegetable fiber, g/day[b]	6.2 (2.4)	6.3 (2.5)	6.3 (2.6)	6.3 (2.5)
Cereal fiber, g/day[b]	4.2 (2.2)	4.3 (2.3)	4.4 (2.4)	4.3 (2.4)
Ratio of total carbohydrate-to-fiber	12.2 (3.4)	12.0 (3.4)	12.0 (3.4)	12.0 (3.4)
Glycemic Index[b]	52.9 (3.3)	52.8 (3.3)	52.9 (3.4)	52.9 (3.7)
Glycemic Load[b]	98.6 (17.5)	98.9 (17.7)	99.6 (17.8)	99.2 (24.5)
Total energy (Kcal/d)[2]	1760 (490)	1753 (490)	1751 (499)	1752 (492)
Total fat intake (% of total energy)[2]	34.0 (5.2)	33.8 (5.3)	33.8 (5.3)	33.8 (5.3)
Total protein intake (% of total energy)[2]	18.1 (3.0)	18.3 (3.0)	18.3 (3.0)	18.1 (3.0)
AHEI[2]	44.9 (9.6)	45.3 (9.7)	45.9 (9.7)	45.5 (9.7)
Race, white, No. (%)	45090 (94.9)	58017 (94.7)	32060 (93.8)	77199 (94.5)
Married, No. (%)	41954 (88.3)	53790 (87.8)	29907 (87.5)	71481 (87.5)
Education				
Registered nurse, No. (%)	336867 (70.9)	46316 (75.6)	25942 (75.9)	60207 (73.7)
Bachelors, No. (%)	9122 (19.2)	11211 (18.3)	5571 (16.3)	14378 (17.6)
Master's or higher, No. (%)	4704 (9.2)	5575 (9.1)	2665 (7.8)	7107 (8.7)
Physical activity (MET-h/week)[2]	12.5 (19.7)	12.4 (19.6)	12.0 (20.7)	12.2 (20.0)
BMI[3]				
BMI < 25 kg/m^2, No. (%)	29601 (62.3)	37065 (60.5)	20747 (60.7)	50159 (61.4)
BMI 25 to <30 kg/m^2, No. (%)	12021 (25.3)	15990 (26.1)	9502 (27.8)	21648 (26.5)

© 2025 Ardisson Korat et al. *JAMA Network Open*.

BMI ≥ 30 kg/m^2, No. (%)	5892 (12.4)	8209 (13.4)	3965 (11.6)	9885 (12.1)
Smoking status				
Never smoker, No. (%)	20336 (42.8)	26221 (42.8)	16064 (47.0)	36026 (44.1)
Past smoker, No. (%)	16297 (34.3)	21197 (34.6)	11655 (34.1)	28184 (34.5)
Current smoker, No. (%)	10880 (22.9)	13846 (22.6)	6460 (18.9)	17482 (21.4)
Postmenopausal hormone use				
Premenopausal	22759 (47.9)	24077 (39.3)	13672 (40.0)	35291 (43.2)
Never used, No. (%)	13874 (29.2)	19482 (31.8)	10903 (31.9)	25243 (30.9)
Former user, No. (%)	5797 (12.2)	8148 (13.3)	4546 (13.3)	10212 (12.5)
Current user, No. (%)	4276 (9.0)	8087 (13.2)	3350 (9.8)	7761 (9.5)
Current aspirin use (%)	33972 (71.5)	43375 (70.8)	24370 (71.3)	58246 (71.3)
Multivitamin use, No. (%)	17247 (36.3)	22606 (36.9)	12749 (37.3)	30389 (37.2)
Hypertension, No. (%)	7697 (16.2)	11028 (18.0)	5674 (16.6)	13397 (16.4)
High cholesterol, No. (%)	2566 (5.4)	3798 (6.2)	2017 (5.9)	4575 (5.6)

AHEI, Alternative Healthy Eating Index; BMI, body mass index; FFQ, food frequency questionnaire; METs, metabolic equivalent; Q, quintile; SD, standard deviation; SSB, sugar-sweetened beverage

a. Summary statistics include age-standardized means and standard deviations for continuous covariates (except for age) and proportions for categorical variables by quintiles of protein intake expressed in percent of total energy intake

b. Mean, SD (all such values)

© 2025 Ardisson Korat et al. *JAMA Network Open*.

eTable 2. Primary Contributors for Each Carbohydrate Variable

Carbohydrate intake variable	Primary dietary contributors
Total carbohydrates	Mashed potatoes, white bread, breakfast cereal, whole grain bread, orange juice, skim milk, apples, bananas, white rice, pizza, punch, pasta, pastries, soda, added sugar, yogurt, peaches, oranges, ice cream, jam, tomato sauce, cake, French fries, candy, corn, pancakes
Carbohydrates from whole grains	Breakfast cereal, whole grain bread, oatmeal/cooked oats, brown rice, popcorn, bran
Carbohydrates from fruits (excluding fruit juice)	Bananas, apples, oranges, raisins, grapefruit, cantaloupe, watermelon, peaches, strawberries, blueberries, processed foods (pies, cereals, yogurt)
Carbohydrates from vegetables (excluding potatoes and legumes)	Corn, tomatoes, carrots, broccoli, lettuce, tomato sauce, squash, cabbage, mixed vegetables, beets, cauliflower, brussels sprouts, pizza, spinach, celery
Carbohydrates from legumes	Beans, peas, mixed vegetables, nuts, peanut butter, processed foods, tofu
Carbohydrates from starchy vegetables	Summed intakes of carbohydrates from potatoes, corn, and yams
Carbohydrates from potatoes	Mashed potatoes, French fries, potato chips, chowder
High-quality carbohydrates	Summed intakes of carbohydrates from fruits (excluding fruit juice), vegetables (excluding potatoes and legumes), whole grains, and legumes
Refined carbohydrates	Mashed potatoes, bread, pasta, white rice, pizza, pastries, soda, added sugar, bread rolls, cake, yogurt, jam, French fries, ice cream, punch, pancakes, potato chips, candy, donuts,
Dietary fiber	Breakfast cereal, apples, whole grain bread, oranges, bananas, peas, tomatoes, carrots, lettuce, broccoli, beans, pizza, pasta, mixed vegetables, nuts, corn, cabbage, oatmeal/oats
Fiber from fruits	Apples, oranges, bananas, orange juice, strawberries, grapefruit, peaches, cantaloupes, prunes, raisins, blueberries, watermelon, fruit in pastries, apple juice, grapefruit juice
Fiber from vegetables	Peas, tomato sauce, carrots, tomatoes, beans, lettuce, broccoli, mixed vegetables, string beans, corn, cabbage, cauliflower, squash, spinach, pizza toppings, Brussels sprouts, yams, celery, beets, mushrooms
Fiber from cereals	Breakfast cereal, whole-grain bread, white bread, pasta, oatmeal/cooked oats, pastries, pizza, popcorn, bran, brown rice, pancakes, white rice
Glycemic Load	Mashed potatoes, white bread, breakfast cereal, whole grain bread, orange juice, bananas, white rice, pizza, pasta, pastries, punch, soda, apples, skim milk, pancakes, added sugar, peaches, jam, fruit juice, carrots, candy, French fries,
Total carbohydrate-to-fiber ratio	Total carbohydrates divided by dietary fiber

© 2025 Ardisson Korat et al. *JAMA Network Open*.

eTable 3. ORs (95% CIs) of Individual Domains of Healthy Aging According to Intake of Carbohydrates In 1984/1986 Among 47,513 Participants in the Nurses' Health Study Without Adjustment for BMI (ORs >1 denote greater odds of healthy aging)

	OR (95% CI) for specified energy increments				
	**Absence of chronic diseases (n=15,056)**	**No memory impairments (n=23,196)**	**No physical function limitations (n=7,300)**	**Good mental status (n=18,204)**	**Healthy Aging (n=3,706)**
**Total Carbohydrates - per 10% energy increment**					
Multivariate model 2[a]	1.00 (0.97, 1.04)	1.04 (1.01, 1.08)	1.08 (1.03, 1.14)	1.07 (1.03, 1.11)	1.17 (1.10, 1.25)
Multivariate model – BMI[b]	1.08 (1.04, 1.11)	1.04 (1.01, 1.07)	1.14 (1.09, 1.20)	1.07 (1.04, 1.11)	1.20 (1.13, 1.27)
**High-quality carbohydrates[c] - per 10% energy increment**					
Multivariate model 2[a]	1.08 (1.04, 1.13)	1.08 (1.04, 1.12)	1.24 (1.77, 1.31)	1.17 (1.12, 1.22)	1.31 (1.22, 1.41)
Multivariate model – BMI[b]	1.11 (1.07, 1.16)	1.10 (1.07, 1.16)	1.29 (1.22, 1.36)	1.21 (1.16, 1.26)	1.37 (1.28, 1.47)
**Refined carbohydrates[d] - per 10% energy increment**					
Multivariate model 2[a]	0.92 (0.88, 0.97)	1.03 (0.98, 1.07)	0.91 (0.85, 0.97)	0.94 (0.90, 0.99)	0.87 (0.80, 0.95)
Multivariate model – BMI[b]	0.99 (0.95, 1.03)	1.00 (0.96, 1.04)	0.96 (0.92, 1.01)	0.94 (0.91, 0.98)	0.92 (0.86, 0.99)
**Carbohydrates from whole grains - per 5% energy increment**					
Multivariate model 2[a]	1.10 (1.04, 1.15)	1.00 (0.96, 1.06)	1.10 (1.03, 1.18)	1.07 (1.02, 1.12)	1.11 (1.01, 1.21)
Multivariate model – BMI[b]	1.15 (1.10, 1.21)	1.03 (0.98, 1.08)	1.20 (1.12, 1.28)	1.12 (1.06, 1.17)	1.20 (1.10, 1.31)
**Carbohydrates from fruits excluding fruit juice - per 5% energy increment**					
Multivariate model 2[a]	1.06 (1.03, 1.09)	1.06 (1.03, 1.10)	1.16 (1.11, 1.21)	1.11 (1.08, 1.15)	1.22 (1.15, 1.28)
Multivariate model – BMI[b]	1.07 (1.04, 1.10)	1.07 (1.04, 1.11)	1.18 (1.13, 1.22)	1.13 (1.09, 1.16)	1.23 (1.17, 1.30)
**Carbohydrates from vegetables excluding potatoes and legumes - per 5% energy increment**					
Multivariate model 2[a]	0.95 (0.88, 1.04)	1.11 (1.02, 1.20)	1.31 (1.18, 1.46)	1.23 (1.14, 1.33)	1.37 (1.20, 1.57)
Multivariate model – BMI[b]	0.93 (0.86, 1.01)	1.14 (1.12, 1.21)	1.28 (1.15, 1.41)	1.24 (1.15, 1.34)	1.36 (1.20, 1.55)
**Carbohydrates from starchy vegetables[e] - per 5% energy increment**					
Multivariate model 2[a]	0.92 (0.87, 0.97)	1.00 (0.98, 1.01)	0.94 (0.87, 1.01)	1.02 (0.97, 1.08)	0.90 (0.82, 0.99)
Multivariate model – BMI[b]	0.93 (0.88, 0.98)	1.03 (1.00, 1.12)	0.94 (0.88, 1.01)	1.02 (0.97, 1.08)	0.90 (0.82, 0.99)
**Carbohydrates from legumes - per 1% energy increment**					
Multivariate model 2[a]	0.99 (0.96, 1.02)	1.00 (0.98, 1.03)	1.03 (0.99, 1.07)	1.02 (0.99, 1.05)	1.06 (1.01, 1.12)
Multivariate model – BMI[b]	1.00 (0.98, 1.03)	1.01 (0.99, 1.04)	1.06 (1.02, 1.10)	1.04 (1.01, 1.07)	1.09 (1.04, 1.15)

BMI, body mass index; CI, confidence interval; METs, metabolic equivalent; OR, odds ratio

a. Multivariate model 2 was adjusted for baseline age (continuous), race (white, other), education (registered nurse, bachelor, or graduate), marital status (married, other), postmenopausal hormone use (pre-menopausal; never, past user, current user), smoking status (never smoked, former smoker, 0.1–14.9, 15.0–29.9, >30 pack-years), alcohol intake (0, 0.1–4.9, 5.0–14.9,

© 2025 Ardisson Korat et al. *JAMA Network Open*.

>15.0g/d), physical activity (<3, 3-8.9, 9-17.9, 18-26.9, >27 METs/week), baseline history of hypertension or hypercholesterolemia (yes, no), aspirin use (never, past, current), multivitamin use (yes, no), total energy intake (kcal/day, quintiles), BMI (averaged 1984 and 1986 (<22.5, 22.5–24.9, 25.0–27.5, 27.5–30.0, 30.0–34.9, >35.0 kg/m^2), and dietary protein.

b. BMI model was adjusted for the covariates in multivariate model 2 excluding BMI

c. High-quality carbohydrates include Carbohydrates from fruits (excluding fruit juice), carbohydrates from vegetables (excluding potatoes and legumes), carbohydrates from whole grains, and carbohydrates from legumes.

d. Refined carbohydrates include carbohydrates from refined grains, potatoes, and added sugars.

e. Carbohydrates from starchy vegetables include carbohydrates from potatoes, corn, and yams.

© 2025 Ardisson Korat et al. *JAMA Network Open*.

eTable 4. ORs (95% CIs) of Healthy Aging (n=3,706) According to Intake of Carbohydrates Cumulatively Averaged Through 2002 or 2006 Among 47,513 Participants in the Nurses' Health Study (ORs >1 denote greater odds of healthy aging)

	Quintile of carbohydrate intake					*P*-trend[c]	OR (95% CI) for 10%-energy increment
	Q1	Q2	Q3	Q4	Q5		
**Total Carbohydrates**							
2002 Cumulative averaged intake							
Healthy ager, No.	634	731	789	798	754		
Median intake (IQR), % energy	42.0 (39.3, 43.7)	47.1 (46.2, 48.0)	50.3 (49.6, 51.1)	53.4 (52.6, 54.3)	58.0 (56.5, 60.3)		
Age-adjusted model	1.00	1.17 (1.04, 1.31)	1.33 (1.19, 1.49)	1.45 (1.29, 1.62)	1.68 (1.49, 1.88)	<.001	1.37 (1.29, 1.45)
Multivariate model 1[a]	1.00	1.11 (0.98, 1.25)	1.20 (1.06, 1.36)	1.27 (1.12, 1.43)	1.36 (1.20, 1.55)	<.001	1.22 (1.14, 1.31)
Multivariate model 2[b]	1.00	1.14 (1.01, 1.29)	1.27 (1.12, 1.44)	1.36 (1.20, 1.55)	1.53 (1.33, 1.76)	<.001	1.32 (1.23, 1.42)
2006 Cumulative averaged intake							
Healthy ager, No	660	779	793	769	705		
Median intake (IQR), % energy	42.2 (39.4, 43.8)	47.2 (46.3, 48.1)	50.4 (49.7, 51.1)	53.5 (52.7, 54.4)	58.0 (56.5, 60.3)		
Age-adjusted model	1.00	1.21 (1.08, 1.35)	1.29 (1.16, 1.44)	1.38 (1.23, 1.54)	1.61 (1.43, 1.80)	<.001	1.32 (1.24, 1.40)
Multivariate model 1[a]	1.00	1.17 (1.04, 1.32)	1.21 (1.07, 1.36)	1.25 (1.10, 1.42)	1.37 (1.20, 1.57)	<.001	1.21 (1.13, 1.29)
Multivariate model 2[b]	1.00	1.21 (1.08, 1.37)	1.27 (1.13, 1.44)	1.34 (1.18, 1.53)	1.53 (1.34, 1.76)	<.001	1.30 (1.21, 1.40)
**High-quality carbohydrates[d]**							
2002 Cumulative averaged intake							
Healthy ager, No	583	702	782	787	852		
Median intake (IQR), % energy	10.0 (8.5, 11.1)	13.5 (12.8, 14.3)	16.5 (15.7, 17.2)	19.9 (18.9, 20.9)	26.2 (23.9, 30.6)		
Age-adjusted model	1.00	1.39 (1.23, 1.56)	1.73 (1.54, 1.94)	2.05 (1.82, 2.30)	3.05 (2.71, 3.43)	<.001	2.01 (1.89, 2.15)
Multivariate model 1[a]	1.00	1.21 (1.07, 1.36)	1.41 (1.25, 1.59)	1.54 (1.36, 1.74)	2.10 (1.85, 2.38)	<.001	1.61 (1.49, 1.73)
Multivariate model 2[b]	1.00	1.20 (1.06, 1.36)	1.39 (1.23, 1.57)	1.53 (1.35, 1.73)	2.08 (1.83, 2.36)	<.001	1.60 (1.48, 1.72)
2006 Cumulative averaged intake							
Healthy ager, No	651	713	723	824			
Median intake (IQR), % energy	10.5 (8.9, 11.5)	14.0 (13.3, 14.7)	16.9 (16.2, 17.6)	20.2 (19.3, 21.2)	26.1 (24, 30.2)		
Age-adjusted model	1.00	1.41 (1.25, 1.58)	1.77 (1.58, 1.99)	2.10 (1.92, 2.40)	3.12 (2.78, 3.48)	<.001	2.08 (1.95, 2.23)
Multivariate model 1[a]	1.00	1.20 (1.09, 1.41)	1.43 (1.28, 1.64)	1.63 (1.41, 1.89)	2.21 (1.91, 2.70)	<.001	1.68 (1.56, 1.81)
Multivariate model 2[b]	1.00	1.20 (1.09, 1.40)	1.39 (1.26, 1.62)	1.53 (1.37, 1.74)	2.17 (1.93, 2.62)	<.001	1.67 (1.55, 1.80)
**Refined carbohydrates[e]**							
2002 Cumulative averaged intake							
Healthy ager, No	687	778	752	802	687		
Median intake (IQR), % energy	17.8 (16.1, 19.0)	21.4 (20.7, 22.1)	24.0 (23.3, 24.6)	26.6 (25.9, 27.4)	30.9 (29.4, 33.3)		
Age-adjusted model	1.00	0.94 (0.84, 1.06)	0.81 (0.72, 0.91)	0.78 (0.70, 0.88)	0.61 (0.54, 0.69)	<.001	0.71 (0.66, 0.76)
Multivariate model 1[a]	1.00	0.94 (0.83, 1.05)	0.84 (0.74, 0.95)	0.81 (0.72, 0.91)	0.68 (0.60, 0.78)	<.001	0.76 (0.70, 0.82)
Multivariate model 2[b]	1.00	0.93 (0.83, 1.05)	0.84 (0.74, 0.95)	0.81 (0.71, 0.92)	0.67 (0.58, 0.78)	<.001	0.74 (0.67, 0.81)
2006 Cumulative averaged intake							

© 2025 Ardisson Korat et al. *JAMA Network Open*.

Healthy ager, No	769	806	747	758	626		
Median intake (IQR), % energy	17.5 (15.8, 18.7)	21.1 (20.3, 21.7)	23.6 (23.0, 24.2)	26.3 (25.5, 27.1)	30.6 (29.1, 33.1)		
Age-adjusted model	1.00	0.88 (0.79, 0.98)	0.74 (0.66, 0.82)	0.69 (0.62, 0.77)	0.53 (0.47, 0.60)	<.001	0.65 (0.60, 0.69)
Multivariate model 1[a]	1.00	0.87 (0.78, 0.98)	0.77 (0.69, 0.87)	0.72 (0.64, 0.81)	0.61 (0.54, 0.70)	<.001	0.71 (0.65, 0.76)
Multivariate model 2[b]	1.00	0.87 (0.77, 0.97)	0.76 (0.67, 0.86)	0.70 (0.62, 0.80)	0.58 (0.51, 0.68)	<.001	0.67 (0.61, 0.74)

BMI, body mass index; CI, confidence interval; IQR, interquintile range; METs, metabolic equivalent; OR, odds ratio; Q, quintile

a. Multivariate model 1 was adjusted for baseline age (continuous), race (white, other), education (registered nurse, bachelor, or graduate), marital status (married, other), postmenopausal hormone use (pre-menopausal; never, past user, current user), smoking status (never smoked; former smoker, 0.1–14.9, 15.0–29.9, >30 pack-years), alcohol intake (0, 0.1–4.9, 5.0–14.9, >15.0g/d), physical activity (<3, 3-8.9, 9-17.9, 18-26.9, >27 METs/week), baseline history of hypertension or hypercholesterolemia (yes, no), aspirin use (never, past, current), multivitamin use (yes, no), BMI (averaged 1984 and 1986; <22.5, 22.5–24.9, 25.0–27.5, 27.5–30.0, 30.0–34.9, >35.0 kg/m^2), and total energy intake (kcal/day, quintiles). Intakes of alcohol and total energy were cumulatively averaged for the respective analyses of 2002 or 2006 cumulatively averaged protein variables.

b. Multivariate model 2 included covariates in multivariate model 1 with additional adjustment for dietary protein.

c. *P*-trend was calculated by assigning median values to each quintile and was treated as a continuous variable.

d. High-quality carbohydrates include Carbohydrates from fruits (excluding fruit juice), carbohydrates from vegetables (excluding potatoes and legumes), carbohydrates from whole grains, and carbohydrates from legumes.

e. Refined carbohydrates include carbohydrates from refined grains, potatoes, and added sugars.

© 2025 Ardisson Korat et al. *JAMA Network Open*.

eTable 5. ORs (95% CIs) of Individual Domains of Healthy Aging According to Intake of Carbohydrates Cumulatively Averaged Through 2002 or 2006 Among 47,513 Participants in the Nurses' Health Study (ORs >1 denote greater odds of healthy aging)

	OR (95% CI) for 10%-energy increment			
	Absence of chronic diseases (n=15,056)	No memory impairments (n=23,196)	No physical function limitations (n=7,300)	Good mental status (n=18,204)
**Total Carbohydrates**				
2002 Cumulative averaged intake				
Age-adjusted model	1.24 (1.20, 1.28)	1.25 (1.22, 1.29)	1.29 (1.23, 1.34)	1.29 (1.25, 1.34)
Multivariate model 1[a]	1.08 (1.04, 1.12)	1.10 (1.06, 1.14)	1.14 (1.08, 1.20)	1.13 (1.09, 1.17)
Multivariate model 2[b]	1.08 (1.03, 1.12)	1.15 (1.11, 1.20)	1.21 (1.15, 1.29)	1.20 (1.15, 1.25)
2006 Cumulative averaged intake				
Age-adjusted model	1.20 (1.16, 1.24)	1.21 (1.17, 1.25)	1.23 (1.18, 1.29)	1.25 (1.21, 1.29)
Multivariate model 1[a]	1.06 (1.02, 1.11)	1.07 (1.03, 1.11)	1.11 (1.05, 1.17)	1.10 (1.06, 1.14)
Multivariate model 2[b]	1.05 (1.01, 1.10)	1.11 (1.07, 1.15)	1.18 (1.12, 1.25)	1.17 (1.12, 1.22)
**High-quality carbohydrates[c]**				
2002 Cumulative averaged intake				
Age-adjusted model	1.44 (1.39, 1.50)	1.52 (1.47, 1.58)	1.93 (1.83, 2.03)	1.68 (1.61, 1.74)
Multivariate model 1[a]	1.17 (1.12, 1.23)	1.24 (1.19, 1.29)	1.52 (1.43, 1.61)	1.37 (1.31, 1.43)
Multivariate model 2[b]	1.18 (1.13, 1.24)	1.23 (1.17, 1.28)	1.50 (1.41, 1.59)	1.35 (1.29, 1.41)
2006 Cumulative averaged intake				
Age-adjusted model	1.49 (1.43, 1.55)	1.61 (1.55, 1.67)	2.00 (1.90, 2.11)	1.76 (1.70, 1.83)
Multivariate model 1[a]	1.23 (1.17, 1.28)	1.32 (1.27, 1.38)	1.59 (1.50, 1.69)	1.45 (1.39, 1.52)
Multivariate model 2[b]	1.24 (1.19, 1.30)	1.31 (1.25, 1.41)	1.58 (1.49, 1.67)	1.43 (1.37, 1.50)
**Refined carbohydrates[d]**				
2002 Cumulative averaged intake				
Age-adjusted model	0.89 (0.86, 0.93)	0.90 (0.86, 0.93)	0.72 (0.68, 0.76)	0.82 (0.79, 0.85)
Multivariate model 1[a]	0.93 (0.89, 0.97)	0.94 (0.90, 0.98)	0.79 (0.74, 0.84)	0.85 (0.81, 0.88)
Multivariate model 2[b]	0.87 (0.83, 0.92)	0.99 (0.94, 1.04)	0.78 (0.73, 0.84)	0.86 (0.82, 0.91)
2006 Cumulative averaged intake				
Age-adjusted model	0.83 (0.80, 0.86)	0.81 (0.78, 0.84)	0.65 (0.62, 0.69)	0.74 (0.72, 0.77)
Multivariate model 1[a]	0.88 (0.84, 0.92)	0.86 (0.82, 0.89)	0.72 (0.68, 0.77)	0.77 (0.74, 0.81)
Multivariate model 2[b]	0.80 (0.76, 0.84)	0.87 (0.82, 0.91)	0.70 (0.65, 0.76)	0.77 (0.73, 0.81)

BMI, body mass index; CI, confidence interval; METs, metabolic equivalent; OR, odds ratio

a. Multivariate model 1 was adjusted for baseline age (continuous), race (white, other), education (registered nurse, bachelor, or graduate), marital status (married, other), postmenopausal hormone use (pre-menopausal; never, past user, current user), smoking status (never smoked; former smoker, 0.1–14.9, 15.0–29.9, >30 pack-years), alcohol intake (0, 0.1–4.9, 5.0–14.9, >15.0g/d), physical activity (<3, 3-8.9, 9-17.9, 18-26.9, >27 METs/week), baseline history of hypertension or hypercholesterolemia (yes, no), aspirin use (never, past, current), multivitamin use (yes, no), BMI (averaged 1984 and 1986; <22.5, 22.5–24.9, 25.0–27.5, 27.5–30.0, 30.0–34.9, >35.0 kg/m^2), and total energy intake (kcal/day, quintiles). Intakes of alcohol and total energy were cumulatively averaged for the respective analyses of 2002 or 2006 cumulatively averaged protein variables.

© 2025 Ardisson Korat et al. *JAMA Network Open*.

b. Multivariate model 2 included covariates in multivariate model 1 with additional adjustment for dietary protein.
c. High-quality carbohydrates include Carbohydrates from fruits (excluding fruit juice), carbohydrates from vegetables (excluding potatoes and legumes), carbohydrates from whole grains, and carbohydrates from legumes.
d. Refined carbohydrates include carbohydrates from refined grains, potatoes, and added sugars.

© 2025 Ardisson Korat et al. *JAMA Network Open*.

eTable 6. ORs (95% CIs) of Healthy Aging (n = 3,706) Assessed in 2014/2016 According to Measures of Carbohydrate Quality in 1984/1986 Among 47,513 Participants in the Nurses' Health Study (ORs >1 denote greater odds of healthy aging)

	Quintile of indices of carbohydrate quality					*P*-trend[d]	OR (95% CI) for interquintile range
	Q1	Q2	Q3	Q4	Q5		
**Glycemic load**							
Healthy ager, No.	618	767	755	788	778		
Median intake (IQR), % energy	77.0 (70.0, 81.5)	90.0 (87.5, 92.5)	98.5 (96.5, 100.4)	107.1 (104.5, 109.5)	120.0 (115.5, 127.0)		
Age-adjusted model	1.00	1.12 (1.00, 1.26)	1.19 (1.06, 1.34)	1.16 (1.04, 1.30)	1.26 (1.13, 1.42)	<.001	1.19 (1.09, 1.30)
Multivariate model 1[a]	1.00	1.05 (0.93, 1.19)	1.10 (0.97, 1.24)	1.02 (0.90, 1.16)	1.06 (0.94, 1.21)	0.53	1.03 (0.90, 1.38)
Multivariate model 2[b]	1.00	1.08 (0.96, 1.22)	1.16 (1.02, 1.32)	1.11 (0.97, 1.27)	1.21 (1.05, 1.40)	0.02	1.16 (1.03, 1.31)
Multivariate model 3[c]	1.00	1.02 (0.90, 1.15)	1.04 (0.92, 1.19)	0.96 (0.83, 1.10)	0.98 (0.84, 1.14)	0.57	0.94 (0.83, 1.08)
**Glycemic index**							
Healthy ager, No	711	769	738	788	700		
Median intake (IQR), % energy	48.8 (47.3, 49.7)	51.4 (50.9, 51.8)	53.0 (52.7, 53.4)	54.6 (54.2, 55.0)	58.8 (56.1, 57.9)		
Age-adjusted model	1.00	0.96 (0.86, 1.07)	0.86 (0.77, 0.96)	0.86 (0.77, 0.97)	0.72 (0.64, 0.80)	<.001	0.76 (0.70, 0.83)
Multivariate model 1[a]	1.00	0.98 (0.88, 1.10)	0.86 (0.77, 0.97)	0.88 (0.79, 0.99)	0.75 (0.66, 0.84)	<.001	0.78 (0.71, 0.86)
Multivariate model 2[b]	1.00	0.99 (0.88, 1.11)	0.88 (0.77, 0.98)	0.90 (0.80, 1.01)	0.76 (0.67, 0.87)	<.001	0.79 (0.72, 0.88)
**Ratio of total carbohydrate-to-fiber**							
Healthy ager, No	706	811	720	775	694		
Median intake (IQR), % energy	8.8 (8.1, 9.3)	10.3 (10.0, 10.7)	11.6 (11.3, 12.0)	13.1 (12.7, 13.6)	16.0 (15.0, 18.0)		
Age-adjusted model	1.00	0.98 (0.88, 1.09)	0.77 (0.69, 0.86)	0.74 (0.66, 0.83)	0.58 (0.51, 0.65)	<.001	0.93 (0.92, 0.95)
Multivariate model 1[a]	1.00	1.04 (0.93, 1.16)	0.84 (0.75, 0.94)	0.82 (0.73, 0.93)	0.70 (0.62, 0.79)	<.001	0.96 (0.94, 0.97)
Multivariate model 2[b]	1.00	1.04 (0.93, 1.17)	0.84 (0.74, 0.95)	0.83 (0.73, 0.94)	0.71 (0.62, 0.81)	<.001	0.96 (0.94, 0.97)

BMI, body mass index; CI, confidence interval; METs, metabolic equivalent; OR, odds ratio; Q, quintile

a. Multivariate model 1 was adjusted for baseline age (continuous), race (white, other), education (registered nurse, bachelor, or graduate), marital status (married, other), postmenopausal hormone use (pre-menopausal; never, past user, current user), smoking status (never smoked, former smoker, 0.1–14.9, 15.0–29.9, >30 pack-years), alcohol intake (0, 0.1–4.9, 5.0–14.9, >15.0g/d), physical activity (<3, 3-8.9, 9-17.9, 18-26.9, >27 METs/week), baseline history of hypertension or hypercholesterolemia (yes, no), aspirin use (never, past, current), multivitamin use (yes, no), total energy intake (kcal/day, quintiles), and BMI (averaged 1984 and 1986; <22.5, 22.5–24.9, 25.0–27.5, 27.5–30.0, 30.0–34.9, >35.0 kg/m^2).

b. Multivariate model 2 included covariates in multivariate model 1 with additional adjustment for dietary protein.

c. Multivariate model 3 included covariates in multivariate model 2 with further adjustment for dietary fiber.

d. *P*-trend was calculated by assigning median values to each quintile and was treated as a continuous variable.

© 2025 Ardisson Korat et al. *JAMA Network Open*.

eTable 7. ORs (95% CIs) of Individual Domains of Healthy Aging According to Intake of Carbohydrates in 1984/1986 Among 47,513 Participants In the Nurses' Health Study With Further Adjustment for Dietary Fiber (ORs >1 denote greater odds of healthy aging)

	OR (95% CI) for specified energy increments				
	Absence of chronic diseases (n=15,056)	No memory impairments (n=23,196)	No physical function limitations (n=7,300)	Good mental status (n=18,204)	Healthy Aging (n=3,706)
**Total Carbohydrates - per 10% energy increment**					
Multivariate model 2[a]	1.00 (0.97, 1.04)	1.04 (1.01, 1.08)	1.08 (1.03, 1.14)	1.07 (1.03, 1.11)	1.17 (1.10, 1.25)
Multivariate model + fiber[b]	0.95 (0.99, 1.00)	0.99 (0.95, 1.03)	0.95 (0.90, 1.03)	0.98 (0.94, 1.03)	1.02 (0.95, 1.11)
**High-quality carbohydrates[c] - per 10% energy increment**					
Multivariate model 2[a]	1.08 (1.04, 1.13)	1.08 (1.04, 1.12)	1.24 (1.77, 1.31)	1.17 (1.12, 1.22)	1.31 (1.22, 1.41)
Multivariate model + fiber[b]	1.01 (0.94, 1.10)	0.94 (0.87, 1.01)	0.99 (0.90, 1.10)	1.04 (0.96, 1.12)	1.08 (0.94, 1.23)
**Refined carbohydrates[d] - per 10% energy increment**					
Multivariate model 2[a]	0.92 (0.88, 0.97)	1.03 (0.98, 1.07)	0.91 (0.85, 0.97)	0.94 (0.90, 0.99)	0.87 (0.80, 0.95)
Multivariate model + fiber[b]	0.94 (0.90, 0.99)	1.06 (1.00, 1.10)	0.98 (0.92, 1.05)	0.99 (0.94, 1.03)	0.95 (0.88, 1.04)
**Carbohydrates from whole grains - per 5% energy increment**					
Multivariate model 2[a]	1.10 (1.04, 1.15)	1.00 (0.96, 1.06)	1.10 (1.03, 1.18)	1.07 (1.02, 1.12)	1.11 (1.01, 1.21)
Multivariate model + fiber[b]	1.06 (1.00, 1.12)	0.93 (0.88, 0.98)	0.94 (0.87, 1.01)	0.96 (0.91, 1.02)	0.92 (0.83, 1.01)
**Carbohydrates from fruits excluding fruit juice - per 5% energy increment**					
Multivariate model 2[a]	1.06 (1.03, 1.09)	1.06 (1.03, 1.10)	1.16 (1.11, 1.21)	1.11 (1.08, 1.15)	1.22 (1.15, 1.28)
Multivariate model + fiber[b]	1.03 (0.99, 1.07)	1.02 (0.98, 1.06)	1.05 (0.99, 1.11)	1.05 (1.01, 1.09)	1.10 (1.03, 1.18)
**Carbohydrates from vegetables excluding potatoes and legumes - per 5% energy increment**					
Multivariate model 2[a]	0.95 (0.88, 1.04)	1.11 (1.02, 1.20)	1.31 (1.18, 1.46)	1.23 (1.14, 1.33)	1.37 (1.20, 1.57)
Multivariate model + fiber[b]	0.78 (0.70, 0.87)	0.95 (0.86, 1.05)	0.95 (0.82, 1.09)	1.00 (0.90, 1.11)	0.94 (0.79, 1.12)
**Carbohydrates from starchy vegetables[e] - per 5% energy increment**					
Multivariate model 2[a]	0.92 (0.87, 0.97)	1.00 (0.98, 1.01)	0.94 (0.87, 1.01)	1.02 (0.97, 1.08)	0.90 (0.82, 0.99)
Multivariate model + fiber[b]	0.91 (0.86, 0.96)	1.05 (1.00, 1.11)	0.91 (0.85, 0.98)	1.00 (0.95, 1.06)	0.87 (0.79, 0.96)
**Carbohydrates from legumes - per 1% energy increment**					
Multivariate model 2[a]	0.99 (0.96, 1.02)	1.00 (0.98, 1.03)	1.03 (0.99, 1.07)	1.02 (0.99, 1.05)	1.06 (1.01, 1.12)
Multivariate model + fiber[b]	0.96 (0.93, 0.99)	0.97 (0.94, 1.00)	0.96 (0.92, 1.00)	0.97 (0.94, 1.00)	0.98 (0.93, 1.03)

BMI, body mass index; CI, confidence interval; METs, metabolic equivalent; OR, odds ratio

a. Multivariate model 2 was adjusted for baseline age (continuous), race (white, other), education (registered nurse, bachelor, or graduate), marital status (married, other), postmenopausal hormone use (pre-menopausal; never, past user, current user), smoking status (never smoked, former smoker, 0.1–14.9, 15.0–29.9, >30 pack-years), alcohol intake (0, 0.1–4.9, 5.0–14.9,

© 2025 Ardisson Korat et al. *JAMA Network Open*.

>15.0g/d), physical activity (<3, 3-8.9, 9-17.9, 18-26.9, >27 METs/week), baseline history of hypertension or hypercholesterolemia (yes, no), aspirin use (never, past, current), multivitamin use (yes, no), total energy intake (kcal/day, quintiles), BMI (averaged 1984 and 1986 (<22.5, 22.5–24.9, 25.0–27.5, 27.5–30.0, 30.0–34.9, >35.0 kg/m^2), and dietary protein.

b. Fiber model was adjusted for the covariates in multivariate model 2 with further adjustment for dietary fiber intake (averaged 1984 and 1986, continuous)
c. High-quality carbohydrates include Carbohydrates from fruits (excluding fruit juice), carbohydrates from vegetables (excluding potatoes and legumes), carbohydrates from whole grains, and carbohydrates from legumes.
d. Refined carbohydrates include carbohydrates from refined grains, potatoes, and added sugars.
e. Carbohydrates from starchy vegetables include carbohydrates from potatoes, corn, and yams.

© 2025 Ardisson Korat et al. *JAMA Network Open*.

eTable 8. ORs (95% CIs) of Individual Domains of Healthy Aging According to Intake of Carbohydrates in 1984/1986 Among 47,513 Participants in the Nurses' Health Study With Additional Adjustment for B-Vitamins and Polyphenols (ORs >1 denote greater odds of healthy aging)

	OR (95% CI) for specified energy increments				
	**Absence of chronic diseases (n=15,056)**	**No memory impairments (n=23,196)**	**No physical function limitations (n=7,300)**	**Good mental status (n=18,204)**	**Healthy Aging (n=3,706)**
	**Total Carbohydrates - per 10% energy increment**				
Multivariate model 1[a]	1.01 (0.98, 1.04)	1.02 (0.98, 1.05)	1.03 (0.99, 1.08)	1.02 (0.99, 1.06)	1.09 (1.03, 1.15)
MV1 + B-vitamins[b]	1.01 (0.98, 1.04)	1.02 (0.98, 1.05)	1.03 (0.99, 1.08)	1.03 (0.99, 1.06)	1.09 (1.03, 1.15)
MV1 + Polyphenols[c]	1.01 (0.98, 1.05)	1.01 (0.98, 1.05)	1.03 (0.98, 1.08)	1.02 (0.99, 1.06)	1.08 (1.02, 1.15)
MV1 + B-vitamins and polyphenols[d]	1.01 (0.98, 1.04)	1.01 (0.98, 1.05)	1.03 (0.98, 1.08)	1.02 (0.99, 1.06)	1.08 (1.02, 1.15)
	**High-quality carbohydrates[e] - per 10% energy increment**				
Multivariate model 1[a]	1.08 (1.03, 1.12)	1.08 (1.04, 1.13)	1.25 (1.18, 1.32)	1.18 (1.13, 1.23)	1.32 (1.23, 1.42)
MV1 + B-vitamins[b]	1.08 (1.03, 1.12)	1.09 (1.05, 1.14)	1.26 (1.19, 1.33)	1.19 (1.14, 1.24)	1.33 (1.24, 1.43)
MV1 + Polyphenols[c]	1.08 (1.04, 1.13)	1.08 (1.03, 1.12)	1.25 (1.18, 1.32)	1.18 (1.13, 1.23)	1.31 (1.22, 1.41)
MV1 + B-vitamins and polyphenols[d]	1.08 (1.04, 1.13)	1.09 (1.04, 1.13)	1.26 (1.19, 1.33)	1.18 (1.14, 1.24)	1.32 (1.23, 1.42)
	**Refined carbohydrates[f] - per 10% energy increment**				
Multivariate model 1[a]	0.96 (0.92, 0.99)	0.99 (0.95, 1.03)	0.90 (0.85, 0.94)	0.92 (0.88, 0.95)	0.86 (0.81, 0.92)
MV1 + B-vitamins[b]	0.96 (0.92, 0.99)	0.99 (0.95, 1.02)	0.89 (0.85, 0.94)	0.92 (0.88, 0.95)	0.86 (0.80, 0.92)
MV1 + Polyphenols[c]	0.96 (0.92, 0.99)	0.99 (0.96, 1.03)	0.90 (0.85, 0.94)	0.92 (0.89, 0.96)	0.87 (0.81, 0.93)
MV1 + B-vitamins and polyphenols[d]	0.96 (0.92, 0.99)	0.99 (0.95, 1.03)	0.89 (0.85, 0.94)	0.92 (0.88, 0.95)	0.86 (0.81, 0.92)
	**Carbohydrates from whole grains - per 5% energy increment**				
Multivariate model 1[a]	1.10 (1.04, 1.15)	1.01 (0.96, 1.06)	1.10 (1.03, 1.18)	1.08 (1.02, 1.13)	1.11 (1.02, 1.21)
MV1 + B-vitamins[b]	1.09 (1.04, 1.15)	1.02 (0.97, 1.07)	1.11 (1.04, 1.19)	1.08 (1.02, 1.14)	1.12 (1.02, 1.22)
MV1 + Polyphenols[c]	1.10 (1.04, 1.15)	1.01 (0.96, 1.06)	1.10 (1.03, 1.18)	1.07 (1.02, 1.13)	1.11 (1.02, 1.21)
MV1 + B-vitamins and polyphenols[d]	1.09 (1.04, 1.15)	1.01 (0.96, 1.06)	1.11 (1.04, 1.19)	1.08 (1.03, 1.13)	1.11 (1.02, 1.21)
	**Carbohydrates from fruits excluding fruit juice - per 5% energy increment**				
Multivariate model 1[a]	1.06 (1.03, 1.09)	1.07 (1.04, 1.10)	1.16 (1.12, 1.21)	1.12 (1.08, 1.15)	1.22 (1.16, 1.28)
MV1 + B-vitamins[b]	1.06 (1.03, 1.09)	1.07 (1.04, 1.10)	1.17 (1.12, 1.22)	1.12 (1.09, 1.16)	1.22 (1.16, 1.29)
MV1 + Polyphenols[c]	1.06 (1.03, 1.10)	1.06 (1.03, 1.09)	1.16 (1.11, 1.21)	1.11 (1.08, 1.15)	1.21 (1.15, 1.28)
MV1 + B-vitamins and polyphenols[d]	1.06 (1.03, 1.10)	1.06 (1.03, 1.10)	1.17 (1.12, 1.22)	1.12 (1.08, 1.15)	1.21 (1.15, 1.28)
	**Carbohydrates from vegetables excluding potatoes and legumes - per 5% energy increment**				
Multivariate model 1[a]	0.95 (0.87, 1.03)	1.12 (1.04, 1.21)	1.33 (1.20, 1.48)	1.26 (1.16, 1.36)	1.41 (1.24, 1.61)
MV1 + B-vitamins[b]	0.95 (0.87, 1.02)	1.13 (1.05, 1.22)	1.35 (1.21, 1.49)	1.27 (1.17, 1.37)	1.41 (1.24, 1.61)
MV1 + Polyphenols[c]	0.95 (0.87, 1.03)	1.11 (1.03, 1.20)	1.33 (1.20, 1.48)	1.25 (1.15, 1.35)	1.38 (1.21, 1.58)
MV1 + B-vitamins and polyphenols[d]	0.95 (0.87, 1.03)	1.11 (1.03, 1.21)	1.34 (1.20, 1.49)	1.25 (1.15, 1.35)	1.38 (1.21, 1.58)

© 2025 Ardisson Korat et al. *JAMA Network Open*.

	**Carbohydrates from starchy vegetables[g] - per 5% energy increment**				
Multivariate model 1[a]	0.92 (0.88, 0.98)	1.00 (0.98, 1.01)	0.94 (0.87, 1.01)	1.02 (0.97, 1.08)	0.90 (0.92, 0.99)
MV1 + B-vitamins[b]	0.93 (0.88, 0.98)	1.06 (1.01, 1.12)	0.93 (0.87, 1.01)	1.02 (0.97, 1.07)	0.90 (0.92, 0.99)
MV1 + Polyphenols[c]	0.92 (0.88, 0.98)	1.06 (1.01, 1.12)	0.94 (0.87, 1.01)	1.02 (0.97, 1.07)	0.90 (0.81, 0.99)
MV1 + B-vitamins and polyphenols[d]	0.92 (0.88, 0.98)	1.06 (1.01, 1.12)	0.94 (0.87, 1.01)	1.02 (0.96, 1.07)	0.89 (0.81, 0.98)
	**Carbohydrates from legumes - per 1% energy increment**				
Multivariate model 1[a]	1.02 (0.99, 1.05)	1.03 (1.01, 1.06)	1.09 (1.05, 1.13)	1.06 (1.03, 1.09)	1.07 (1.02, 1.12)
MV1 + B-vitamins[b]	0.99 (0.96, 1.02)	1.01 (0.98, 1.04)	1.04 (1.00, 1.08)	1.03 (0.99, 1.06)	1.07 (1.02, 1.12)
MV1 + Polyphenols[c]	0.99 (0.96, 1.02)	1.01 (0.98, 1.03)	1.04 (0.98, 1.08)	1.03 (1.00, 1.06)	1.07 (1.02, 1.12)
MV1 + B-vitamins and polyphenols[d]	0.99 (0.96, 1.02)	1.01 (0.98, 1.04)	1.04 (1.00, 1.08)	1.03 (1.00, 1.06)	1.07 (1.02, 1.12)

BMI, body mass index; CI, confidence interval; METs, metabolic equivalent; OR, odds ratio

a. Multivariate model 1 was adjusted for baseline age (continuous), race (white, other), education (registered nurse, bachelor, or graduate), marital status (married, other), postmenopausal hormone use (pre-menopausal; never, past user, current user), smoking status (never smoked, former smoker, 0.1–14.9, 15.0–29.9, >30 pack-years), alcohol intake (0, 0.1–4.9, 5.0–14.9, >15.0g/d), physical activity (<3, 3-8.9, 9-17.9, 18-26.9, >27 METs/week), baseline history of hypertension or hypercholesterolemia (yes, no), aspirin use (never, past, current), multivitamin use (yes, no), total energy intake (kcal/day, quintiles), and BMI (averaged 1984 and 1986; <22.5, 22.5–24.9, 25.0–27.5, 27.5–30.0, 30.0–34.9, >35.0 kg/m^2), and dietary protein.
b. B vitamin model was adjusted for the covariates in multivariate model 1 and levels of B-vitamins
c. B vitamin model was adjusted for the covariates in multivariate model 1 and polyphenol levels
d. B vitamin model was adjusted for the covariates in multivariate model 1 and levels of B-vitamins and polyphenol levels
e. High-quality carbohydrates include Carbohydrates from fruits (excluding fruit juice), carbohydrates from vegetables (excluding potatoes and legumes), carbohydrates from whole grains, and carbohydrates from legumes.
f. Refined carbohydrates include carbohydrates from refined grains, potatoes, and added sugars.
g. Carbohydrates from starchy vegetables include carbohydrates from potatoes, corn, and yams.

© 2025 Ardisson Korat et al. *JAMA Network Open*.

eTable 9. ORs (95% CIs) of Individual Domains of Healthy Aging According to Intake of Carbohydrate in 1984/1986 Among 47,513 Participants in the Nurses' Health Study (ORs >1 denote greater odds of healthy aging)

	OR (95% CI) for specified energy increments			
	Absence of chronic diseases (n=15,056)	No memory impairments (n=23,196)	No physical function limitations (n=7,300)	Good mental status (n=18,204)
**Total Carbohydrates - per 10% energy increment**				
Age-adjusted model	1.12 (1.09, 1.15)	1.12 (1.09, 1.15)	1.14 (1.09, 1.18)	1.14 (1.11, 1.17)
Multivariate model 1[a]	1.01 (0.98, 1.04)	1.02 (0.98, 1.05)	1.03 (0.99, 1.08)	1.02 (0.99, 1.06)
Multivariate model 2[b]	1.00 (0.97, 1.04)	1.04 (1.01, 1.08)	1.08 (1.03, 1.14)	1.07 (1.03, 1.11)
**High-quality carbohydrates[c] - per 10% energy increment**				
Age-adjusted model	1.29 (1.24, 1.34)	1.31 (1.27, 1.36)	1.57 (1.50, 1.65)	1.42 (1.37, 1.48)
Multivariate model 1[a]	1.08 (1.03, 1.12)	1.08 (1.04, 1.13)	1.25 (1.18, 1.32)	1.18 (1.13, 1.23)
Multivariate model 2[b]	1.08 (1.04, 1.13)	1.08 (1.04, 1.12)	1.24 (1.17, 1.31)	1.17 (1.12, 1.22)
**Refined carbohydrates[d] - per 10% energy increment**				
Age-adjusted model	0.93 (0.89, 0.96)	0.94 (0.91, 0.97)	0.83 (0.79, 0.87)	0.89 (0.86, 0.92)
Multivariate model 1[a]	0.96 (0.92, 0.99)	0.99 (0.95, 1.03)	0.90 (0.85, 0.94)	0.92 (0.88, 0.95)
Multivariate model 2[b]	0.92 (0.88, 0.97)	1.03 (0.98, 1.07)	0.91 (0.85, 0.97)	0.94 (0.90, 0.99)
**Carbohydrates from whole grains - per 5% energy increment**				
Age-adjusted model	1.33 (1.27, 1.40)	1.22 (1.17, 1.28)	1.42 (1.33, 1.51)	1.32 (1.26, 1.38)
Multivariate model 1[a]	1.10 (1.04, 1.15)	1.01 (0.96, 1.06)	1.10 (1.03, 1.18)	1.08 (1.02, 1.13)
Multivariate model 2[b]	1.10 (1.04, 1.15)	1.01 (0.96, 1.06)	1.10 (1.03, 1.18)	1.07 (1.02, 1.12)
**Carbohydrates from fruits excluding fruit juice - per 5% energy increment**				
Age-adjusted model	1.19 (1.16, 1.23)	1.21 (1.18, 1.25)	1.34 (1.30, 1.39)	1.27 (1.23, 1.30)
Multivariate model 1[a]	1.06 (1.03, 1.09)	1.07 (1.04, 1.10)	1.16 (1.12, 1.21)	1.12 (1.08, 1.15)
Multivariate model 2[b]	1.06 (1.03, 1.09)	1.06 (1.03, 1.10)	1.16 (1.11, 1.21)	1.11 (1.08, 1.15)
**Carbohydrates from vegetables excluding potatoes and legumes - per 5% energy increment**				
Age-adjusted model	1.02 (0.95, 1.10)	1.24 (1.16, 1.33)	1.57 (1.43, 1.72)	1.36 (1.27, 1.46)
Multivariate model 1[a]	0.95 (0.87, 1.03)	1.12 (1.04, 1.21)	1.33 (1.20, 1.48)	1.26 (1.16, 1.36)
Multivariate model 2[b]	0.95 (0.88, 1.04)	1.11 (1.02, 1.20)	1.31 (1.18, 1.46)	1.23 (1.14, 1.33)
**Carbohydrates from starchy vegetables[e] - per 5% energy increment**				
Age-adjusted model	0.86 (0.82, 0.91)	0.99 (0.94, 1.03)	0.81 (0.76, 0.87)	0.93 (0.89, 0.98)
Multivariate model 1[a]	0.92 (0.88, 0.98)	1.00 (0.98, 1.01)	0.94 (0.87, 1.01)	1.02 (0.97, 1.08)
Multivariate model 2[b]	0.92 (0.87, 0.97)	1.00 (0.98, 1.01)	0.94 (0.87, 1.01)	1.02 (0.97, 1.08)

© 2025 Ardisson Korat et al. *JAMA Network Open*.

**Carbohydrates from legumes - per 1% energy increment**				
Age-adjusted model	1.02 (0.99, 1.05)	1.03 (1.01, 1.06)	1.09 (1.05, 1.13)	1.06 (1.03, 1.09)
Multivariate model 1[a]	0.99 (0.96, 1.02)	1.01 (0.98, 1.04)	1.04 (1.00, 1.08)	1.03 (1.00, 1.06)
Multivariate model 2[b]	0.99 (0.96, 1.02)	1.00 (0.98, 1.03)	1.03 (0.99, 1.07)	1.02 (0.99, 1.05)

BMI, body mass index; CI, confidence interval; METs, metabolic equivalent; OR, odds ratio

a. Multivariate model 1 was adjusted for baseline age (continuous), race (white, other), education (registered nurse, bachelor, or graduate), marital status (married, other), postmenopausal hormone use (pre-menopausal; never, past user, current user), smoking status (never smoked, former smoker, 0.1–14.9, 15.0–29.9, >30 pack-years), alcohol intake (0, 0.1–4.9, 5.0–14.9, >15.0g/d), physical activity (<3, 3-8.9, 9-17.9, 18-26.9, >27 METs/week), baseline history of hypertension or hypercholesterolemia (yes, no), aspirin use (never, past, current), multivitamin use (yes, no), total energy intake (kcal/day, quintiles), and BMI (averaged 1984 and 1986; <22.5, 22.5–24.9, 25.0–27.5, 27.5–30.0, 30.0–34.9, >35.0 kg/m^2).
b. Multivariate model 2 included covariates in multivariate model 1 with additional adjustment for dietary protein.
c. High-quality carbohydrates include Carbohydrates from fruits (excluding fruit juice), carbohydrates from vegetables (excluding potatoes and legumes), carbohydrates from whole grains, and carbohydrates from legumes.
d. Refined carbohydrates include carbohydrates from refined grains, potatoes, and added sugars.
e. Carbohydrates from starchy vegetables include carbohydrates from potatoes, corn, and yams.

© 2025 Ardisson Korat et al. *JAMA Network Open*.

eTable 10. ORs (95% CIs) of Individual Domains of Healthy Aging According to Intake of Dietary Fiber in 1984/1986 Among 47,513 Participants in the Nurses' Health Study (ORs >1 denote greater odds of healthy aging)

	OR (95% CI) per 1-SD increment[c]			
	Absence of chronic diseases (n=15,056)	No memory impairments (n=23,196)	No physical function limitations (n=7,300)	Good mental status (n=18,204)
**Total fiber**				
Age-adjusted model	1.15 (1.13, 1.18)	1.18 (1.15, 1.20)	1.27 (1.24, 1.31)	1.22 (1.20, 1.25)
Multivariate model 1[a]	1.05 (1.02, 1.07)	1.07 (1.04, 1.09)	1.15 (1.12, 1.19)	1.11 (1.08, 1.13)
Multivariate model 2[b]	1.05 (1.03, 1.07)	1.06 (1.04, 1.09)	1.15 (1.11, 1.18)	1.10 (1.08, 1.13)
**Fruit fiber**				
Age-adjusted model	1.12 (1.10, 1.15)	1.14 (1.11, 1.16)	1.20 (1.17, 1.23)	1.17 (1.14, 1.19)
Multivariate model 1[a]	1.05 (1.03, 1.07)	1.05 (1.03, 1.08)	1.11 (1.08, 1.15)	1.08 (1.05, 1.10)
Multivariate model 2[b]	1.05 (1.03, 1.07)	1.05 (1.03, 1.07)	1.11 (1.08, 1.15)	1.08 (1.06, 1.11)
**Vegetable fiber**				
Age-adjusted model	1.02 (1.00, 1.04)	1.07 (1.05, 1.09)	1.14 (1.11, 1.17)	1.11 (1.09, 1.13)
Multivariate model 1[a]	0.99 (0.97, 1.02)	1.04 (1.02, 1.06)	1.09 (1.06, 1.13)	1.08 (1.06, 1.10)
Multivariate model 2[b]	1.00 (0.98, 1.02)	1.04 (1.01, 1.06)	1.09 (1.06, 1.12)	1.07 (1.05, 1.10)
**Cereal fiber**				
Age-adjusted model	1.16 (1.14, 1.18)	1.11 (1.09, 1.13)	1.18 (1.15, 1.21)	1.13 (1.11, 1.15)
Multivariate model 1[a]	1.06 (1.04, 1.08)	1.01 (0.99, 1.04)	1.06 (1.03, 1.09)	1.02 (1.03, 1.09)
Multivariate model 2[b]	1.06 (1.04, 1.08)	1.01 (0.98, 1.03)	1.06 (1.03, 1.09)	1.02 (1.00, 1.05)

BMI, body mass index; CI, confidence interval; METs, metabolic equivalent; OR, odds ratio; SD, standard deviation

a. Multivariate model 1 was adjusted for baseline age (continuous), race (white, other), education (registered nurse, bachelor, or graduate), marital status (married, other), postmenopausal hormone use (pre-menopausal; never, past user, current user), smoking status (never smoked, former smoker, 0.1–14.9, 15.0–29.9, >30 pack-years), alcohol intake (0, 0.1–4.9, 5.0–14.9, >15.0g/d), physical activity (<3, 3-8.9, 9-17.9, 18-26.9, >27 METs/week), baseline history of hypertension or hypercholesterolemia (yes, no), aspirin use (never, past, current), multivitamin use (yes, no), total energy intake (kcal/day, quintiles), and BMI (averaged 1984 and 1986; <22.5, 22.5–24.9, 25.0–27.5, 27.5–30.0, 30.0–34.9, >35.0 kg/m^2).

b. Multivariate model 2 included covariates in multivariate model 1 with additional adjustment for dietary protein.

c. Fiber intake variable SDs in grams per day were: total fiber, 4.5; fruit fiber 2.3; vegetable fiber, 2.4; cereal fiber, 2.2.

© 2025 Ardisson Korat et al. *JAMA Network Open*.

eTable 11. ORs (95% CIs) of Individual Domains of Healthy Aging According to Indices of Carbohydrate Quality in 1984/1986 Among 47,513 Participants in the Nurses' Health Study (ORs >1 denote greater odds of healthy aging)

	OR (95% CI) per interquintile range increment			
	**Absence of chronic diseases (n=15,056)**	**No memory impairments (n=23,196)**	**No physical function limitations (n=7,300)**	**Good mental status (n=18,204)**
**Glycemic load**				
Age-adjusted model	1.13 (1.08, 1.19)	1.15 (1.09, 1.20)	1.12 (1.05, 1.20)	1.16 (1.10, 1.22)
Multivariate model 1[a]	0.97 (0.91, 1.03)	1.00 (0.94, 1.06)	0.96 (0.89, 1.04)	1.00 (0.94, 1.06)
Multivariate model 2[b]	0.94 (0.87, 1.00)	1.05 (0.98, 1.12)	1.02 (0.93, 1.12)	1.08 (1.01, 1.15)
Multivariate model 3[c]	0.87 (0.80, 0.93)	0.97 (0.90, 1.04)	0.85 (0.77, 0.94)	0.96 (0.89, 1.03)
**Glycemic index**				
Age-adjusted model	0.87 (0.83, 0.92)	0.89 (0.85, 0.93)	0.76 (0.71, 0.81)	0.86 (0.82, 0.90)
Multivariate model 1[a]	0.88 (0.84, 0.93)	0.93 (0.88, 0.97)	0.79 (0.73, 0.85)	0.89 (0.84, 0.94)
Multivariate model 2[b]	0.85 (0.81, 0.90)	0.94 (0.89, 0.99)	0.79 (0.73, 0.85)	0.91 (0.86, 0.96)
**Ratio of total carbohydrate-to-fiber**				
Age-adjusted model	0.96 (0.95, 0.97)	0.96 (0.95, 0.96)	0.93 (0.92, 0.94)	0.95 (0.94, 0.96)
Multivariate model 1[a]	0.98 (0.97, 0.99)	0.98 (0.97, 0.98)	0.96 (0.95, 0.96)	0.97 (0.96, 0.97)
Multivariate model 2[b]	0.98 (0.97, 0.98)	0.98 (0.97, 0.98)	0.95 (0.94, 0.96)	0.97 (0.96, 0.97)

BMI, body mass index; CI, confidence interval; METs, metabolic equivalent; OR, odds ratio

a. Multivariate model 1 was adjusted for baseline age (continuous), race (white, other), education (registered nurse, bachelor, or graduate), marital status (married, other), postmenopausal hormone use (pre-menopausal, never, past user, current user), smoking status (never smoked, former smoker, 0.1–14.9, 15.0–29.9, >30 pack-years), alcohol intake (0, 0.1–4.9, 5.0–14.9, >15.0g/d), physical activity (<3, 3-8.9, 9-17.9, 18-26.9, >27 METs/week), baseline history of hypertension or hypercholesterolemia (yes, no), aspirin use (never, past, current), multivitamin use (yes, no), total energy intake (kcal/day, quintiles). Intakes of alcohol and total energy were cumulatively averaged for the respective analyses of 2002 or 2006 cumulatively averaged carbohydrate variables.
b. Multivariate model 1 was additionally adjusted for BMI (averaged 1984 and 1986; <22.5, 22.5–24.9, 25.0–27.5, 27.5–30.0, 30.0–34.9, >35.0 kg/m^2).
c. Multivariate model 2 included covariates in multivariate model 2 with additional adjustment for dietary protein.
d. Multivariate model 3 included covariates in multivariate model 2 with further adjustment for dietary fiber.

© 2025 Ardisson Korat et al. *JAMA Network Open*.

eTable 12. ORs (95% CIs) of Healthy Aging Associated With Isocaloric Substitution of Carbohydrate (Total, High-Quality, or From Whole Grains) for Other Macronutrients Modeled in 5%-Energy Increments in 47,513 Participants in the Nurses' Health Study (ORs >1 denote greater odds of healthy aging)

	OR (95% CI) 5%-energy increment from carbohydrates[a,b]				
	Healthy Aging (n=3,706)	Absence of chronic diseases (n=15,056)	No memory impairments (n=23,196)	No physical function limitations (n=7,300)	Good mental status (n=18,204)
			**Total Carbohydrates**		
Total protein	0.88 (0.82, 0.94)	1.01 (0.97, 1.05)	0.96 (0.93, 1.00)	0.90 (0.85, 0.94)	0.92 (0.89, 0.96)
Animal protein	0.93 (0.88, 0.99)	1.03 (1.00, 1.08)	0.99 (0.95, 1.02)	0.96 (0.91, 1.00)	0.96 (0.93, 0.99)
Plant protein	0.63 (0.51, 0.78)	0.75 (0.66, 0.85)	0.77 (0.68, 0.88)	0.56 (0.47, 0.66)	0.71 (0.62, 0.81)
Total fat	1.07 (1.03, 1.11)	0.98 (0.96, 1.00)	0.99 (0.97, 1.02)	1.03 (1.00, 1.06)	1.01 (0.99, 1.03)
Saturated fat	0.92 (0.81, 1.05)	0.89 (0.82, 0.96)	0.99 (0.92, 1.06)	0.91 (0.82, 1.01)	0.98 (0.90, 1.05)
PUFA	0.82 (0.70, 0.97)	0.81 (0.73, 0.89)	0.86 (0.78, 0.94)	0.72 (0.64, 0.82)	0.89 (0.81, 0.97)
Trans fat[c]	1.09 (1.02, 1.15)	1.03 (0.99, 1.06)	0.99 (0.96, 1.02)	1.06 (1.01, 1.11)	1.00 (0.97, 1.04)
			**High-quality carbohydrates[d]**		
Refined carbohydrates[e]	1.16 (1.11, 1.21)	1.04 (1.02, 1.07)	1.03 (1.01, 1.06)	1.12 (1.09, 1.16)	1.09 (1.08, 1.12)
Total protein	1.04 (0.97, 1.13)	1.06 (1.01, 1.11)	1.03 (0.99, 1.07)	1.02 (0.96, 1.10)	1.02 (0.98, 1.08)
Animal protein	1.08 (1.01, 1.16)	1.07 (1.03, 1.12)	0.99 (0.96, 1.03)	1.07 (1.01, 1.13)	1.04 (1.00, 1.09)
Plant protein	0.94 (0.73, 1.20)	0.81 (0.70, 0.94)	0.85 (0.74, 0.98)	0.77 (0.64, 0.94)	0.93 (0.80, 1.08)
Total fat	1.15 (1.10, 1.20)	1.01 (0.99, 1.04)	1.02 (1.00, 1.05)	1.09 (1.06, 1.13)	1.07 (1.04, 1.09)
Saturated fat	0.94 (0.85, 1.12)	0.90 (0.84, 0.97)	1.00 (0.93, 1.08)	0.96 (0.86, 1.07)	1.02 (0.94, 1.10)
PUFA	1.01 (0.85, 1.21)	0.88 (0.79, 0.97)	0.96 (0.86, 1.06)	0.87 (0.76, 0.99)	1.05 (0.95, 1.17)
Trans fat[c]	1.10 (1.03, 1.14)	1.03 (0.99, 1.07)	0.98 (0.95, 1.01)	1.08 (1.03, 1.13)	1.02 (0.98, 1.05)

BMI, body mass index; CI, confidence interval; METs, metabolic equivalent; OR, odds ratio; PUFA, polyunsaturated fat

a. The isocaloric substitutions can be interpreted as the association of increasing the calories contributed by a given dietary carbohydrate variable by 5% energy per day while decreasing the corresponding calories contributed by dietary protein, dietary fat, or other dietary carbohydrate variables on the odds of healthy aging.
b. All models adjusted for baseline age (continuous), race (white, other), education (registered nurse, bachelor, or graduate), marital status (married, other), postmenopausal hormone use (pre-menopausal; never, past user, current user), smoking status (never smoked, former smoker, 0.1–14.9, 15.0–29.9, >30 pack-years), alcohol intake (0, 0.1–4.9, 5.0–14.9, >15.0g/d), physical activity (<3, 3-8.9, 9-17.9, 18-26.9, >27 METs/week); BMI (averaged 1984 and 1986; <22.5, 22.5–24.9, 25.0–27.5, 27.5–30.0, 30.0–34.9, >35.0 kg/m^2), baseline history of hypertension or hypercholesterolemia (yes, no); aspirin use (never, past, current); multivitamin use (yes, no) and total energy intake (kcal/day, continuous).
c. The OR (95% CI) for trans fat replacement are expressed in 1% calories per day increment.
d. High-quality carbohydrates include Carbohydrates from fruits (excluding fruit juice), carbohydrates from vegetables (excluding potatoes and legumes), carbohydrates from whole grains, and carbohydrates from legumes.
e. Refined carbohydrates include carbohydrates from refined grains, potatoes, and added sugars.

© 2025 Ardisson Korat et al. *JAMA Network Open*.

eTable 13. ORs (95% CIs) of Healthy Aging According to Baseline (1984/1986) Total Carbohydrate Intake and Glycemic Load Stratified by GI and AHEI Among 47,513 Participants in the Nurses' Health Study[a]

	Absence of chronic diseases	No memory impairments	No physical function limitations	Good mental status	Healthy Aging	P-interaction
			**Total Carbohydrates[c]**			
Age in 1984[b]						
> 48 years	1.02 (0.97, 1.08)	1.01 (0.96, 1.05)	1.01 (0.91, 1.12)	0.97 (0.92, 1.02)	1.03 (0.89, 1.20)	0.54
≤ 48 years	1.00 (0.96, 1.05)	1.03 (0.98, 1.08)	1.04 (0.99, 1.09)	1.07 (1.02, 1.12)	1.10 (1.03, 1.17)	
Baseline BMI						
≥ 25 kg/m²	1.00 (0.94, 1.06)	0.99 (0.94, 1.05)	1.01 (0.92, 1.12)	1.01 (0.96, 1.07)	1.04 (0.90, 1.20)	0.59
< 25 kg/m²	1.01 (0.97, 1.05)	1.03 (0.99, 1.07)	1.03 (0.98, 1.09)	1.03 (0.99, 1.07)	1.09 (1.03, 1.17)	
Baseline Glycemic Index[b]						
≥ median	0.97 (0.92, 1.02)	0.98 (0.93, 1.02)	0.99 (0.92, 1.05)	0.98 (0.94, 1.03)	1.02 (0.94, 1.11)	.002
< median	1.06 (1.01, 1.11)	1.06 (1.01, 1.11)	1.12 (1.05, 1.20)	1.09 (1.04, 1.14)	1.21 (1.12, 1.32)	
Baseline Dietary Fiber[b]						
≥ median	1.06 (1.00, 1.12)	1.02 (0.97, 1.07)	1.08 (1.00, 1.16)	1.02 (0.97, 1.08)	1.15 (1.05, 1.26)	<.001
< median	0.94 (0.89, 0.99)	0.98 (0.94, 1.03)	0.90 (0.84, 0.96)	0.97 (0.93, 1.02)	0.92 (0.85, 1.00)	
Baseline AHEI[b]						
≥ median	1.04 (0.99, 1.10)	1.02 (0.97, 1.07)	1.06 (0.98, 1.13)	1.04 (0.99, 1.09)	1.11 (1.02, 1.21)	0.15
< median	0.98 (0.93, 1.02)	1.00 (0.96, 1.05)	0.98 (0.92, 1.04)	1.00 (0.96, 1.04)	1.03 (0.95, 1.11)	
			**Glycemic Load[d]**			
Age in 1984[2]						
> 48 years	0.99 (0.90, 1.09)	1.01 (0.93, 1.09)	0.91 (0.76, 1.08)	0.94 (0.86, 1.03)	0.94 (0.72, 1.22)	0.48
≤ 48 years	0.96 (0.89, 1.03)	1.01 (0.94, 1.09)	0.98 (0.89, 1.06)	1.05 (0.98, 1.14)	1.05 (0.94, 1.17)	
Baseline BMI						
≥ 25 kg/m²	0.95 (0.85, 1.06)	0.97 (0.88, 1.06)	0.94 (0.79, 1.12)	0.98 (0.86, 1.08)	1.06 (0.82, 1.35)	0.42
< 25 kg/m²	0.97 (0.91, 1.04)	1.01 (0.95, 1.09)	0.96 (0.88, 1.05)	1.00 (0.94, 1.08)	1.02 (0.91, 1.14)	
Baseline Glycemic Index[b]						
≥ median	0.93 (0.85, 1.02)	0.92 (0.84, 1.00)	0.94 (0.79, 1.12)	0.94 (0.86, 1.03)	1.03 (0.88, 1.42)	<.001
< median	1.11 (1.01, 1.22)	1.11 (1.02, 1.30)	1.21 (1.06, 1.37)	1.18 (1.07, 1.29)	1.34 (1.14, 1.57)	
Baseline Dietary Fiber[b]						
≥ median	1.06 (0.97, 1.16)	1.01 (0.93, 1.10)	1.00 (0.89, 1.13)	1.00 (0.91, 1.09)	1.10 (0.95, 1.28)	<.001
< median	0.86 (0.79, 0.93)	0.96 (0.89, 1.03)	0.82 (0.73, 0.91)	0.93 (0.86, 1.01)	0.85 (0.73, 0.98)	
Baseline AHEI[b]						
≥ median	1.04 (0.95, 1.14)	1.03 (0.95, 1.13)	1.04 (0.92, 1.17)	1.05 (0.96, 1.15)	1.10 (0.95, 1.28)	0.29
< median	0.92 (0.85, 1.00)	0.99 (0.92, 1.06)	0.92 (0.82, 1.02)	0.97 (0.90, 1.05)	0.99 (0.87, 1.14)	

AHEI, Alternative Healthy Eating Index; BMI, body mass index; CI, Confidence Interval; METs, metabolic equivalent; OR, odds ratio;

© 2025 Ardisson Korat et al. *JAMA Network Open*.

a. All models adjusted for baseline age (continuous), race (white, other), education (registered nurse, bachelor, or graduate), marital status (married, other), postmenopausal hormone use (pre-menopausal; never, past user, current user), smoking status (never smoked; former smoker, 0.1–14.9, 15.0–29.9, >30 pack-years), alcohol intake (0, 0.1–4.9, 5.0–14.9, >15.0g/d), physical activity (<3, 3-8.9, 9-17.9, 18-26.9, >27 METs/week); BMI (averaged 1984 and 1986; <22.5, 22.5–24.9, 25.0–27.5, 27.5–30.0, 30.0–34.9, >35.0 kg/m^2), baseline history of hypertension or hypercholesterolemia (yes, no); aspirin use (never, past, current); multivitamin use (yes, no) and total energy intake (kcal/day, continuous). The OR is per 5%-energy increment for total carbohydrates and per interquintile range for Glycemic Load.
b. Median participant age in 1984 was 48.0 years; median; glycemic index for 1984/1986 was 53.0; median AHEI averaged for 1984/1986 was 44.2, median dietary fiber intake averaged for 1984/1986 was 16.0 grams per day.
c. The OR (95% CI) for total carbohydrate intake are expressed in 10%-energy increments.
d. The OR (95% CI) for dietary glycemic load are expressed in interquintile range increments.

© 2025 Ardisson Korat et al. *JAMA Network Open*.

eTable 14. ORs (95% CIs) of Healthy Aging (n = 3,706) Assessed in 2014/2016 According to the Dietary Glycemic Load Cross-Classified by Total Dietary Fiber Levels Assessed in 1984/1986 Among 47,513 Participants in the Nurses' Health Study
(ORs >1 denote greater odds of healthy aging)

	ORs (95% CI) for comparing high vs. low levels of the dietary GL[a]				
	**Absence of chronic diseases (n=15,056)**	**No memory impairments (n=23,196)**	**No physical function limitations (n=7,300)**	**Good mental status (n=18,204)**	**Healthy Aging (n=3,706)**
Low GL/Low fiber[b]	1.00 (ref)	1.00 (ref)	1.00 (ref)	1.00 (ref)	1.00 (ref)
High GL/Low fiber[b]	0.95 (0.90, 1.02)	0.98 (0.92, 1.05)	0.88 (0.80, 0.95)	0.97 (0.91, 1.03)	0.91 (0.81, 1.01)
Low GL/High fiber[b]	1.06 (1.00, 1.13)	1.03 (0.97, 1.10)	1.15 (1.06, 1.25)	1.13 (1.07, 1.21)	1.22 (1.09, 1.36)
High GL/High fiber[b]	1.07 (1.01, 1.13)	1.09 (1.03, 1.16)	1.21 (1.12, 1.31)	1.16 (1.09, 1.23)	1.29 (1.17, 1.42)

BMI, body mass index; CI, confidence interval; GL, glycemic load; METs, metabolic equivalent; OR, odds ratio

a. Multivariate model 1 was adjusted for baseline age (continuous), race (white, other), education (registered nurse, bachelor, or graduate), marital status (married, other), postmenopausal hormone use (pre-menopausal; never, past user, current user), smoking status (never smoked; former smoker, 0.1–14.9, 15.0–29.9, >30 pack-years), alcohol intake (0, 0.1–4.9, 5.0–14.9, >15.0g/d), physical activity (<3, 3-8.9, 9-17.9, 18-26.9, >27 METs/week), baseline history of hypertension or hypercholesterolemia (yes, no), aspirin use (never, past, current), multivitamin use (yes, no), BMI (averaged 1984 and 1986; <22.5, 22.5–24.9, 25.0–27.5, 27.5–30.0, 30.0–34.9, >35.0 kg/m^2), dietary protein, and total energy intake (kcal/day, quintiles).

b. Median dietary glycemic load for 1984/1986 was 98.5, median dietary fiber intake averaged for 1984/1986 was 16.0 grams per day.

© 2025 Ardisson Korat et al. *JAMA Network Open*.

© 2025 Ardisson Korat et al. *JAMA Network Open*.
