# Supplementary material for: Dietary Carbohydrate Intake, Carbohydrate Quality, and Healthy Aging in Women
Source: JAMA Netw Open. 2025 May 16;8(5):e2511056. doi: 10.1001/jamanetworkopen.2025.11056 (PMC12084844; doi:10.1001/jamanetworkopen.2025.11056)
Supplement: Supplement 2. — Data Sharing Statement [file jamanetwopen-e2511056-s002.pdf]

Data Sharing Statement

Ardisson Korat. Dietary Carbohydrate Intake, Carbohydrate Quality, and Healthy Aging in Women. *JAMA Netw Open*. Published May 16, 2025.
doi:10.1001/jamanetworkopen.2025.11056

Data
Data available: No

Additional Information
Explanation for why data not available: Data described in the manuscript, code book, and analytic code will not be made available because of participant confidentiality and privacy concerns. Further information including the procedures to obtain and access data from the Nurses' Health Study is described at https://www.nurseshealthstudy.org/researchers (contact e-mail: nhsaccess@channing.harvard.edu).
